# Selenoprotein H Functions as a PPARα Coactivator to Link Selenium Homeostasis to Hepatic Lipid Metabolism and Protect against Steatohepatitis

**DOI:** 10.1002/advs.202519563

**Published:** 2026-02-08

**Authors:** Yuwei Zhang, Yuchen Wang, Binbin Li, Xin Li, Chenyu Liu, Yanhao Chen, Cheng Tian, Dongmei Wang, Xiaosong Gu, Chunping Jiang, Yuda Wei, Qiurong Ding

**Affiliations:** ^1^ CAS Key Laboratory of Nutrition Metabolism and Food Safety Shanghai Institute of Nutrition and Health University of Chinese Academy of Sciences Chinese Academy of Sciences P. R. China; ^2^ Department of Pathology Linyi People's Hospital Shandong China; ^3^ Jinan Microecological Biomedicine Shandong Laboratory Jinan China; ^4^ Department of Gastrointestinal Surgery Affiliated Changzhou No.2 People's Hospital of Nanjing Medical University The Third Affiliated Hospital of Nanjing Medical University Changzhou Medical Center Nanjing Medical University Changzhou China; ^5^ Division of Hepatobiliary and Transplantation Surgery Department of General Surgery Nanjing Drum Tower Hospital the Affiliated Hospital of Medical School Nanjing University Nanjing China; ^6^ Department of Hepatobiliary and Pancreatic Surgery The Second Affiliated Hospital of Fujian Medical University Quanzhou Fujian China; ^7^ Department of Laboratory Medicine Linyi People's Hospital Shandong China; ^8^ Shanghai Key Laboratory of Reproductive Medicine Shanghai China

**Keywords:** fatty acid oxidation (FAO), metabolic dysfunction‐associated steatohepatitis (MASH), selenium, selenoprotein

## Abstract

Selenium is an essential trace element whose dysregulation is associated with diverse disease risks; however, its specific role in hepatic metabolism remains poorly defined. Here we delineate a novel selenium‐selenoprotein H (SELENOH)‐PPARα signaling axis that is critical for hepatic lipid homeostasis. We first uncovered a global impairment of selenoprotein translation as a key feature of metabolic dysfunction‐associated steatohepatitis (MASH) in human patients and mouse models. Both dietary selenium supplementation and genetically rescuing selenoprotein biosynthesis attenuated MASH pathology, establishing a causal link. Through a targeted screen, we pinpointed SELENOH as the key hepatoprotective selenoprotein governing hepatic fatty acid oxidation (FAO). Diverging from the canonical redox functions of selenoproteins, SELENOH operates as a scaffolding coactivator for the nuclear receptor PPARα. SELENOH binds to ligand‐activated PPARα and orchestrates the assembly and chromatin recruitment of the PPARα‐P300 transactivation complex to drive FAO gene expression. This nexus is disrupted in MASH livers due to SELENOH deficiency but is reconstituted by selenium supplementation. These findings altogether define selenium homeostasis as a fundamental regulator of nuclear receptor function and unveil promising therapeutic avenues for MASH.

## Graphical abstract

Our study identifies selenium deficiency as a hallmark of MASH pathogenesis. Dietary selenium supplementation enhances hepatic fatty acid oxidation (FAO) and attenuates MASH progression by activating the PPARα pathway via selenoprotein H (SELENOH). This selenium‐SELENOH‐PPARα nexus redefines the functional scope of selenoproteins, moving from redox chemistry to nuclear receptor coactivation, highlighting the therapeutic potential of selenium supplementation in MASH treatment.

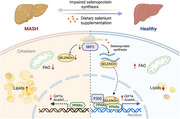

## 1 Introduction

Selenium is an essential micronutrient incorporated into selenoproteins as selenocysteine (Sec), a selenium containing amino acid [1, 2]. The biological effects of selenium are largely mediated by selenoproteins. To date, twenty‐five selenoproteins have been identified in humans, and their well‐characterized functions mainly involve redox reactions [3], such as those catalyzed by glutathione peroxidases (GPXs), thioredoxin reductases (TXNRDs), and methionine‐R‐sulfoxide reductase 1 (MSRB1) [3]. The majority of selenoproteins exhibit thioredoxin or thioredoxin‐like folds, strongly suggesting redox‐related functions [4]. In some selenoproteins (e.g., selenoprotein M [SELENOM], SELENOH, SELENOT, SELENOW, SELENOV), Sec is typically located at the predicted catalytic position, which contains a “redox box” motif (CXXU, where C represents cysteine, X any amino acid, and U selenocysteine) [5]. Despite these insights, the functions of most selenoproteins remain uncharacterized, and their precise substrates have yet to be identified. Furthermore, it is unclear whether selenoproteins have additional roles independent of the redox activity.

The selenium status of an individual is assessed by various biomarkers such as total serum selenium and circulating selenoproteins (such as selenoprotein P from liver and GPX3 from kidney) [6, 7, 8]. Selenium deficiency may lead to insufficient expression and impaired function of one or more selenoproteins, potentially contributing to disease pathogenesis [2]. Growing clinical evidence indicates an association between low selenium status and chronic liver diseases, such as hepatitis B and C virus infection, alcoholic liver diseases, as well as advanced liver fibrosis [9, 10, 11, 12, 13, 14]. These findings underscore the potential role of selenium and selenoproteins in maintaining liver functions.

Metabolic dysfunction‐associated steatotic liver disease (MASLD) has emerged as a global epidemic, characterized by lipid accumulation in more than 5% of hepatocytes (hepatic steatosis). 25∼30% of MASLD patients will develop liver injury, inflammation and fibrosis, a condition defined as metabolic dysfunction‐associated steatohepatitis (MASH) [15, 16]. Chronic steatosis is a main process driving MASH, resulting in hepatocyte stress and subsequent liver inflammation and fibrosis. Therapeutic strategies that reduce hepatocyte lipid content, such as triglyceride synthesis inhibitors [17] and peroxisome proliferator‐activated receptors (PPARs) agonists [18], enable hepatocyte to cope with cellular stress and eventually ameliorate liver inflammation and fibrosis. However, current treatments for MASH remain limited, with only two FDA‐approved agents—resmetirom, a thyroid hormone receptor‐β agonist, and semaglutide, a glucagon‐like peptide‑1 receptor agonist—available [19].

Previous studies have suggested that selenium plays a critical role in liver lipid metabolism, although its exact function remains elusive [20, 21, 22]. Diet‐induced selenium deficiency in pigs resulted in generally reduced hepatic lipid levels, presumably due to inhibited lipid synthesis [20]. Under selenium‐deficient conditions, significant alterations in lipid metabolites were observed in mouse liver, with no consistent direction of changes reported for specific lipid species [21]. Another study showed increased liver lipid peroxidation, along with elevated levels of some long‐chain acylcarnitines and various phosphatidylcholines in mice with a suboptimal selenium supply [22]. Altogether, these data from different metabolomic analyses in animal livers with diet‐induced selenium deficiency have not yet reached a definitive conclusion. In contrast, selenium supplementation [23, 24], or co‐supplementation with zinc [25], consistently displayed significant effects on reducing liver lipid accumulation in mouse or rat models of fatty liver. Although inhibited lipid synthesis gene expression was observed in AML12 cells [23] and liver tissues [24] after selenium supplementation, the exact mechanisms by which selenium influences liver lipid metabolism in both physiological and pathological conditions remain to be further characterized.

In this study, similar other chronic liver conditions, we have detected reduced selenium levels in the livers of individuals with MASH. Further investigation has unveiled notable impairment of the hepatic selenoprotein translational machinery, leading to compromised selenoprotein synthesis. Interventions involving dietary selenium supplementation or restoration of selenoprotein synthesis activity through genetic methods have significantly ameliorated hepatic lipid accumulation and provided protection against MASH. Specifically, through screening of 13 selenoproteins with relatively high expression in liver, we further identified selenoprotein H (SELENOH) as a key regulator of hepatic fatty acid oxidation (FAO) through its interaction with activated peroxisome proliferator‐activated receptor alpha (PPARα), thereby modulating MASH progression. Notably, the function of SELENOH in regulating lipid metabolism has been proved to be independent of its redox enzymatic activity. These findings altogether illuminate specific and previously overlooked mechanisms of selenium in regulating hepatic lipid metabolism and highlight selenium supplementation as a potential treatment for MASH.

## 2 Results

### 2.1 Low Selenium Status Is Associated with Human and Mouse MASH Development

**FIGURE 1:**
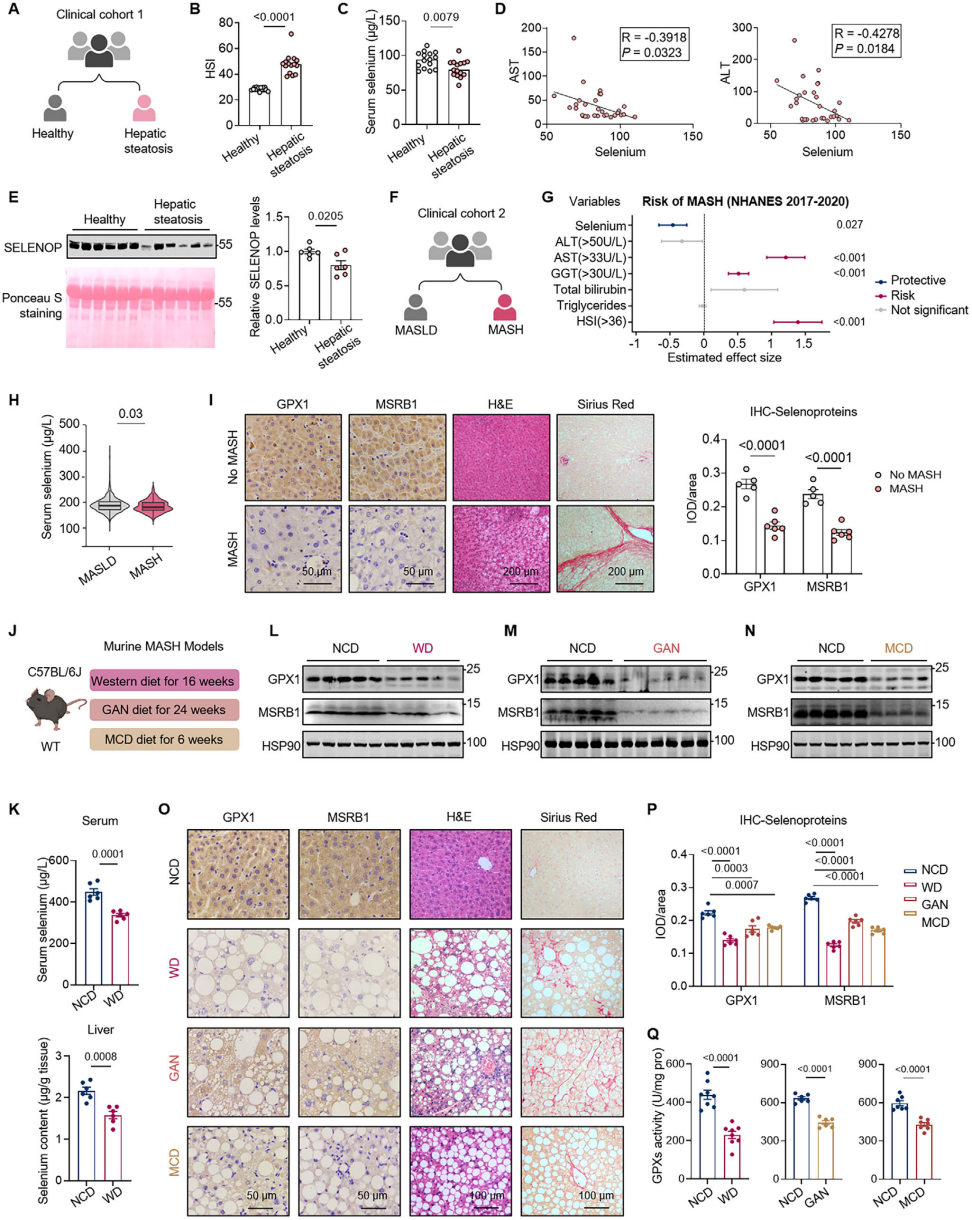
A selenium‐insufficient state in human and mouse MASH. (A) Grouping of the clinical cohort 1, including healthy controls (n = 15) and patients with hepatic steatosis (n = 15). (B) Hepatic steatosis index (HSI) of healthy controls (HSI < 30) and patients with hepatic steatosis (HSI > 36). (C) Selenium levels in the serum samples from clinical cohort 1. (D) Scatter plots showing the linear regression analysis between selenium levels and AST/ALT levels in the serum samples from clinical cohort 1. (E) SELENOP levels in the serum samples from clinical cohort 1. Ponceau S staining is used as a control for protein loading. (F) Grouping of the clinical cohort 2 from National Health and Nutrition Examination Survey (NHANES) 2017–March 2020 dataset, including patients with MASLD (n = 1033) and patients with MASH (n = 271). (G) The analysis of MASH risk in clinical cohort 2 by multi‐variate logistic regression analysis. (H) Selenium levels in the serum samples from clinical cohort 2. (I) Representative GPX1, MSRB1, H&E and Sirius Red staining of liver sections from patients with No‐MASH (n = 5) or MASH (n = 6). (J) Schematic illustration of murine MASH models. (K) Selenium levels in serum and livers from mice fed a normal chow diet (NCD) or western diet (WD), high sugar drink plus CCl_4_ injection per week for 16 weeks. n = 6 per group. (L) Protein levels of GPX1 and MSRB1 in the livers from mice fed NCD (n = 5) or WD (n = 5) for 16 weeks. (M) Protein levels of GPX1 and MSRB1 in the livers from mice fed NCD (n = 5) or Gubra amylin NASH diet (GAN) (n = 6) for 24 weeks. (N) Protein levels of GPX1 and MSRB1 in the livers from mice fed NCD (n = 5) or methionine/choline deficient diet (MCD) (n = 4) for 6 weeks. (O) Representative GPX1, MSRB1, H&E and Sirius Red staining of liver sections from animals as indicated. n = 6 per group. (P) The quantification of selenoprotein GPX1 and MSRB1 intensity based on the immunohistochemistry analysis shown in (O). (Q) Total GPXs activity in liver tissue from animals as indicated. Values are mean ± SEM. The unpaired Student's *t*‐test (B, C, E, I, K, and Q), Mann‐Whitney test (H) and one‐way ANOVA with post hoc Bonferroni multiple‐comparison test (P) were used for statistical analysis.

To investigate the role of selenium in the progression of human MASLD, we examined selenium levels in serum from human subjects with or without hepatic steatosis (15 vs. 15), classified by the hepatic steatosis index (HSI) [26] (Figure 1A,B). Serum selenium levels were significantly lower in patients with liver steatosis than in the healthy controls (Figure 1C). Additionally, serum selenium levels were negatively correlated with serum aspartate aminotransferase (AST) and alanine aminotransferase (ALT) levels, which are markers indicating the severity of liver damage (Figure 1D). Liver is known as the major source of serum selenium by secreting selenoprotein P (SELENOP) [6]. Consistently, we also observed a significant reduction in serum SELENOP levels in patients with hepatic steatosis, reflecting a possibly low‐selenium state in liver (Figure 1E). We then analyzed data from the National Health and Nutrition Examination Survey (NHANES) database to interrogate the relationship between serum selenium level and MASH development (Figure 1F). The multi‐variate logistic regression analysis suggested that the serum selenium level was an independent protective factor for MASH (OR: 0.63, 95% CI: 0.42–0.93, *p* = 0.027), while the elevated levels of AST, gamma‐glutamyl transferase (GGT) and HSI score were independent risk factors (Figure 1G). Participants with MASH also displayed significantly reduced serum selenium levels compared to those with MASLD (Figure 1H). In addition, immunochemistry analysis of liver sections from MASH patients demonstrated decreased levels of selenoproteins including glutathione peroxidase 1 (GPX1) and methionine sulfoxide reductase B1 (MSRB1), which are most sensitive to selenium status (Figure 1I). These data suggest that selenium levels decrease in human subjects with MASH‐features, primarily characterized by reduced liver selenoprotein expression and serum selenium levels.

To validate our findings from the clinical cohorts, we first measured selenium levels in serum and liver of mice fed a MASH‐inducing diet [Western diet (WD)] (Figure 1J). The results showed a significant decrease in selenium levels in MASH animals (Figure 1K). Consistently, the levels of selenoproteins such as GPX1 and MSRB1, as well as the total GPXs activity, were significantly reduced in MASH livers (Figure 1L,O–Q). Moreover, similar downregulation in selenoprotein expression and total GPXs activity were observed in two other murine MASH models induced by the Gubra AMLN (GAN) diet or methionine choline deficient (MCD) diet (Figure 1M–Q). Differently, the levels of two housekeeping selenoproteins GPX4 and TXNRD1 remained unchanged, in line with previously reported selenoprotein hierarchy upon selenium restriction [3, 27] (Figure S1A). Notably, in a MASH resolution model, switching to a normal chow diet (NCD) after 24 weeks of GAN diet feeding led to rapid MASH resolution, characterized by reduced ALT levels, decreased serum total cholesterol (TC) and hepatic triglyceride (TG) content, regression of fibrosis, alongside a recovery in selenoprotein expression (Figure S1B–G). Taken together, these results demonstrate that the low selenium status in both serum and liver is strongly associated with MASH progression.

### 2.2 MASH Liver Exhibits Impaired Selenoprotein Synthesis

**FIGURE 2:**
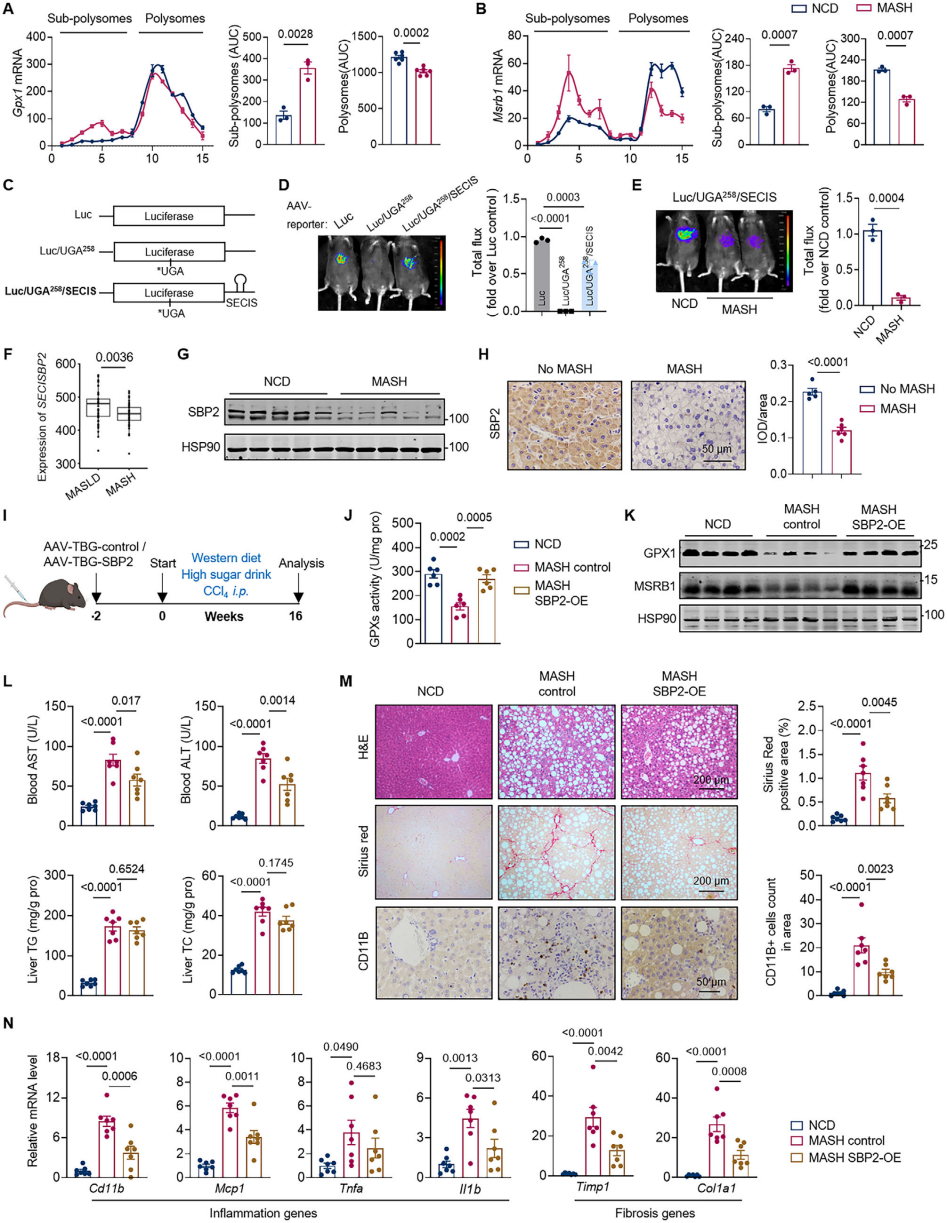
MASH liver exhibits impaired selenoprotein synthesis. (A and B) Total RNA was extracted from the sucrose gradient fractions. RT‐qPCR analysis of distribution of Gpx1 and Msrb1 mRNAs in livers from NCD and WD‐induced MASH mice. n = 3 per group. (C) Schematic illustration of AAV constructs used to assess selenocysteine insertion in vivo. (D) Representative images and normalized quantification of bioluminescent imaging after injection with AAV expressing indicated luciferase reporters in NCD mice. n = 3 per group. (E) Representative images and normalized quantification of bioluminescent imaging after injection with AAV expressing luciferase/UGA^258^/SECIS in NCD and WD‐induced MASH mice. n = 3 per group. (F) *SECISBP2* mRNA expression in FPKM from GSE167523 [MASLD (n = 51) and MASH (n = 47)]. (G) Protein levels of SBP2 in livers from NCD and WD‐induced MASH mice. n = 5 per group. (H) Representative SBP2 staining of liver sections from patients with No‐MASH (n = 5) or MASH (n = 6). Scale bars, 50 µm. (I) Schematic of the experimental design: *Secisbp2* overexpression in WD‐induced MASH mice. (J) Total GPXs activity in liver tissue from animals as indicated. n = 6 per group. (K) Protein levels of GPX1 and MSRB1 in the livers from animals as indicated. n = 4 per group. (L) Blood AST and ALT levels, and hepatic TG and TC levels of animals as indicated. n = 7 per group. (M) Representative H&E, Sirius Red and CD11B staining of liver sections. (N) Relative mRNA levels of genes related to hepatic inflammation and fibrosis. n = 7 per group. Values are mean ± SEM. The unpaired Student's *t*‐test (A, B, E, and H), wilcoxon test (F) and one‐way ANOVA with post hoc Bonferroni multiple‐comparison test (D, J, L–N) were used for statistical analysis.

Our clinical and animal data have revealed a dysregulation of selenium homeostasis in MASH subjects, marked by low selenium level and diminished hepatic selenoprotein expression. We next investigated the underlying causes of affected selenoprotein expression in MASH subjects. Contrary to protein levels, the mRNA levels of selenoproteins in MASH livers are upregulated (Figure S2A), suggesting specific mechanisms in modulating selenoprotein expression beyond transcriptional regulation. We then investigated the translational efficiency of selenoproteins using polysome profiling analysis. The results exhibited a significant decline in the translation rate of selenoproteins such as GPX1 and MSRB1 in MASH livers, indicating impaired selenoprotein synthesis (Figure 2A,B).

Selenoprotein translation relies on the recoding of in‐frame UGA as selenocysteine (Sec), a process mediated by the *cis*‐acting Sec insertion sequence (SECIS) element and special *trans*‐acting protein factors (Figure S2B). To assay the efficiency of this UGA‐Sec recoding, we utilized a Sec incorporation reporter system [28]. In this system, the codon at position 258 in the luciferase coding region was replaced with UGA, followed by a GPX1 SECIS element that directs the recoding of this UGA into Sec, enabling full‐length luciferase translation (Figure 2C). The reporter cassette was cloned into an adeno‐associated virus (AAV) vector under the control of liver‐specific thyroxine‐binding globulin (TBG) promoter to create a tool for liver‐specific interrogation of Sec incorporation. The specificity of this tool was confirmed by demonstrating that the resulting luciferase activity was dependent on both the SECIS element and the UGA codon (Figure 2D). After administering this AAV reporter to mice on diets with 0 ppm and 0.2 ppm selenium, we found that this reporter effectively responds to changes in selenium supply and serves as a reliable indicator of Sec incorporation in vivo (Figure S2C–G). We next injected AAV‐reporter and performed in vivo imaging in MASH animals, which revealed a significant decrease in luciferase activity (Figure 2E), further supporting impaired selenoprotein synthesis during MASH progression.

Selenoprotein synthesis requires a specific set of proteins, in which two unique *trans*‐acting factors are essential: SECIS binding protein 2 (SBP2) and Sec‐specific translation elongation factor (eEFSec) [29] (Figure S2B). We first examined the expression levels of *SECISBP2* and *EEFSEC* using public RNA‐seq data (GSE167523) of individuals with MASLD or MASH. The results showed significantly reduced *SECISBP2* expression in MASH patients, with no change in *EEFSEC* expression (Figure 2F and S2H). We next examined SBP2 levels in liver samples from the above‐mentioned MASH patients and animal models, and observed consistent results, revealing significantly lower SBP2 levels in the MASH groups compared to the control groups (Figure 2G,H and S2I). Interestingly, a previous study showed that SBP2 with a naturally occurring point mutation discovered in humans results in preservation of the synthesis of essential selenoproteins but a decrease in the synthesis of nonessential ones, [30] similar to the hierarchical expression pattern of selenoproteins observed in our MASH model. Taken together, these findings imply that the reduction in the SBP2 level may be a key contributor to the decreased selenoproteins expression observed in MASH subjects.

We further overexpressed SBP2 under the TBG promoter using AAV vectors. After 16 weeks of MASH induction (Figure 2I), SBP2 overexpression significantly restored hepatic selenoprotein expression and the total GPXs activity in livers of MASH mice (Figure 2J,K). Correspondingly, serum AST and ALT levels were significantly decreased, with hepatic lipid accumulation and liver weight also exhibiting a decreasing trend (Figure 2L and S2J). Moreover, SBP2 overexpression attenuated liver inflammation and fibrosis, as indicated by the decreases in the CD11B staining and Sirius red staining, as well as the reduction in the inflammatory and fibrotic gene expression (Figure 2M,N). These results altogether demonstrate that restoring the selenoprotein biosynthesis by SBP2 overexpression markedly protects against MASH.

### 2.3 Selenium Supplementation Alleviates MASH Progression

**FIGURE 3:**
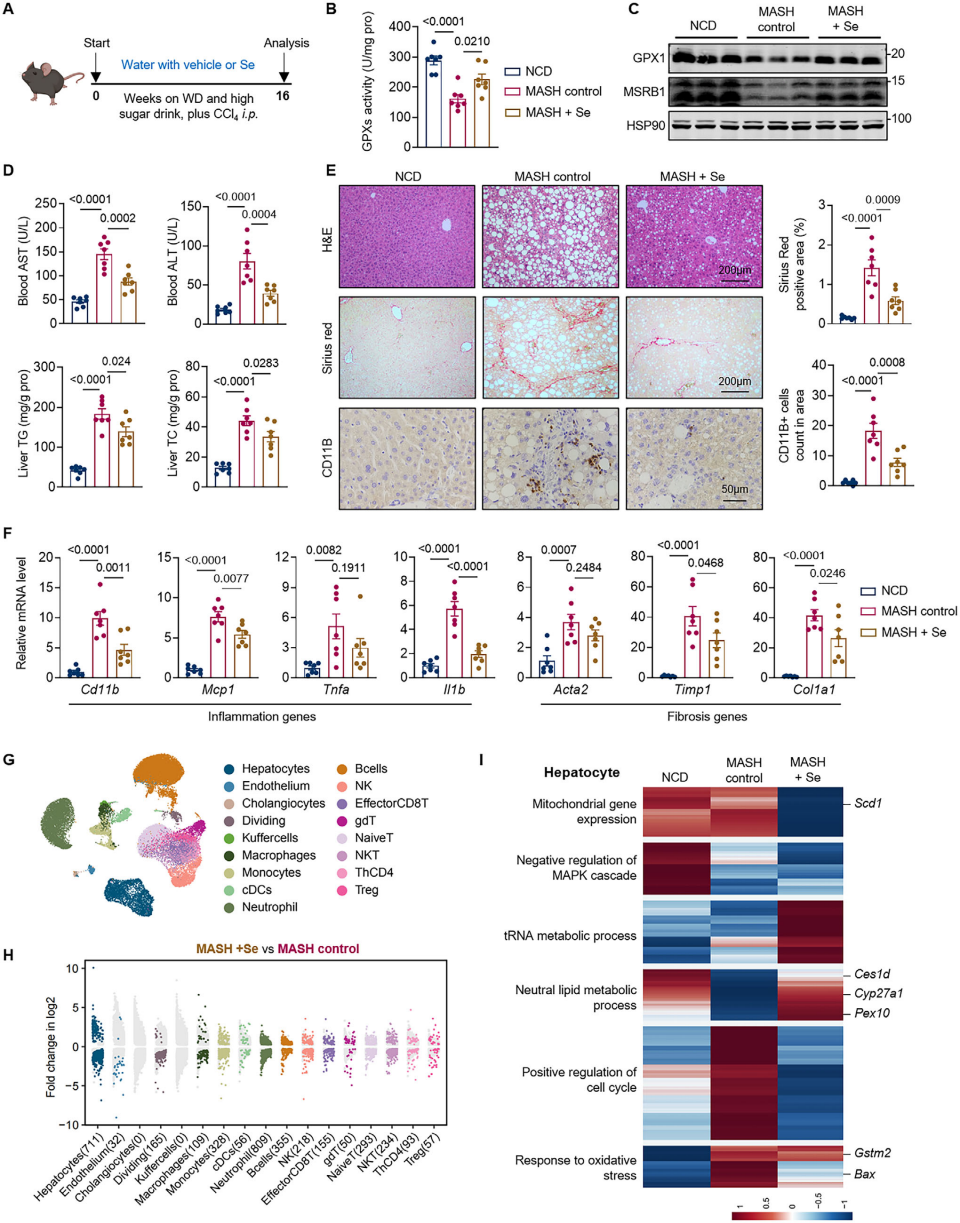
Dietary selenium supplementation alleviates MASH progression. (A) Schematic of the experimental design: selenium intervention in WD‐induced MASH mice. (B) Total GPXs activity in liver tissue from animals as indicated. n = 7 per group. (C) Protein levels of GPX1 and MSRB1 in the livers from animals as indicated. n = 3 per group. (D) Blood AST and ALT levels, and hepatic TG and TC levels of animals as indicated. n = 7 per group. (E) Representative H&E, Sirius Red and CD11B staining of liver sections. (F) Relative mRNA levels of genes related to hepatic inflammation and fibrosis. n = 7 per group. (G) Uniform Manifold Approximation and Projection (UMAP) plot of all cells in single cell RNA‐seq data. (H) Dot plot showing the fold changes of genes in each cell type. Genes with positive values have higher expression levels in MASH + Se group, while those with negative values have higher expression levels in MASH control group. Genes with adjusted *p* value < 0.01 are shown with indicated colors, while genes with insignificant changes between groups are shown in grey dots. The number in parenthesis indicates the number of significantly changed genes. (I) Heatmap showing the unsupervised clusters of genes highly expressed in hepatocytes. Red denotes higher scaled expression levels, while blue denotes lower scaled expression levels. Values are mean ± SEM. The one‐way ANOVA with post hoc Bonferroni multiple‐comparison test (B, D–F) was used for statistical analysis.

Dietary supplementation of selenium is able to increase selenoprotein abundance in individuals with low selenium status [31]. To investigate whether selenium supplementation could restore selenoprotein expression in MASH livers, WT mice were fed with either selenium‐supplemented water or plain water during MASH induction (Figure 3A). Selenium treatment (8 ppm in drinking water) was maintained at concentrations within the safety margin established by previous studies demonstrating no adverse effects in mice [32]. Notably, selenium supplementation significantly increased selenium levels, increased Sec incorporation efficiency and enhanced selenoprotein abundance in MASH livers (Figure 3B,C and S3A,B). Selenium markedly reduced liver weights, with little effects on body weight in MASH mice (Figure S3C). Hepatic TG and TC contents, as well as serum AST and ALT levels were significantly reduced in selenium intervention group, indicating an improvement in the MASH‐related liver steatosis and injury (Figure 3D). Meanwhile, mice treated with selenium exhibited fewer inflammatory cell infiltration and reduced liver fibrosis (Figure 3E,F).

Additionally, we also investigated whether selenium supplementation could aid in MASH resolution and fibrosis regression. After MASH establishing, mice were transitioned to normal chow diet with or without selenium supplementation (Figure S3D). Selenium treatment enhanced selenoprotein expression (Figure S3E) and accelerated MASH resolution, with significant improvements in liver injury, steatosis and fibrosis (Figure S3F–J). These results collectively underscored the effectiveness of selenium supplementation in safeguarding against MASH development and promoting MASH resolution.

Recent studies have reported novel functions of selenoproteins within a wide variety of cell types, including regulation of hepatocyte apoptosis, immune cell differentiation and promotion of neuronal proliferation [32, 33, 34]. To better understand the effects of selenium supplementation on different cell types in liver niches, we performed single‐cell RNA sequencing (scRNA‐seq) of liver tissues from different groups in selenium treatment experiment. A total of 17 cell types were identified, with a mass of genes exhibiting significant alterations across diverse cell populations in liver tissue following selenium supplementation, indicating a broad effect of selenium to multiple cell types (Figure 3G,H and S4A,B). Remarkably, hepatocytes displayed transcriptomic alterations with the greatest magnitudes among all cell populations (Figure 3H). In the hepatocyte population, unsupervised clustering of differentially expressed genes (DEGs) showed two clusters exhibiting a notable reversal following selenium supplementation (Figure 3I). Genes in these clusters were enriched in GO terms including neutral lipid metabolic process and positive regulation of cell cycle, respectively (Figure 3I). Remarkably, pathway analysis demonstrated that genes downregulated in hepatocytes from MASH livers were assigned to multiple lipid metabolic process, and selenium supplementation led to a significant reversal of these genes, suggesting relevant effects of selenium in regulating lipid metabolism (Figure S4C,D). To be noted, selenium supplementation may also affect functions of other cell types in liver niches such as macrophages, of which signatures of lipid associated macrophages (LAMs) were significantly attenuated after selenium treatment (Figure S4E). Whether the clearance of LAMs was directly caused by selenium treatment or consequently followed by the improvement in lipid metabolism in liver tissues warrants further investigation. Taken together, we find that dietary selenium supplementation ameliorates MASH, primarily by modulating lipid metabolism in hepatocytes.

### 2.4 Selenoprotein H Regulates Fatty Acid Oxidation and MASH Progression

The biological effects of selenium are largely carried out by selenoproteins. Building on our findings that demonstrated a strong association between selenium and MASH, we next sought to determine which selenoprotein critically regulates MASH development. Our results indicated that selenium supplementation was able to restore selenoprotein expression, such as GPX1 and MSRB1, which was supposed to account for the mitigation of MASH progression. However, contradictory findings have been made by previous reports with regard to the role of these selenoproteins in liver metabolism. For example, selenoprotein MSRB1 deficiency increases hepatic oxidative stress and exacerbates hepatotoxicity [35], whereas GPX1 depletion represses liver inflammation and fibrosis [36].

**FIGURE 4:**
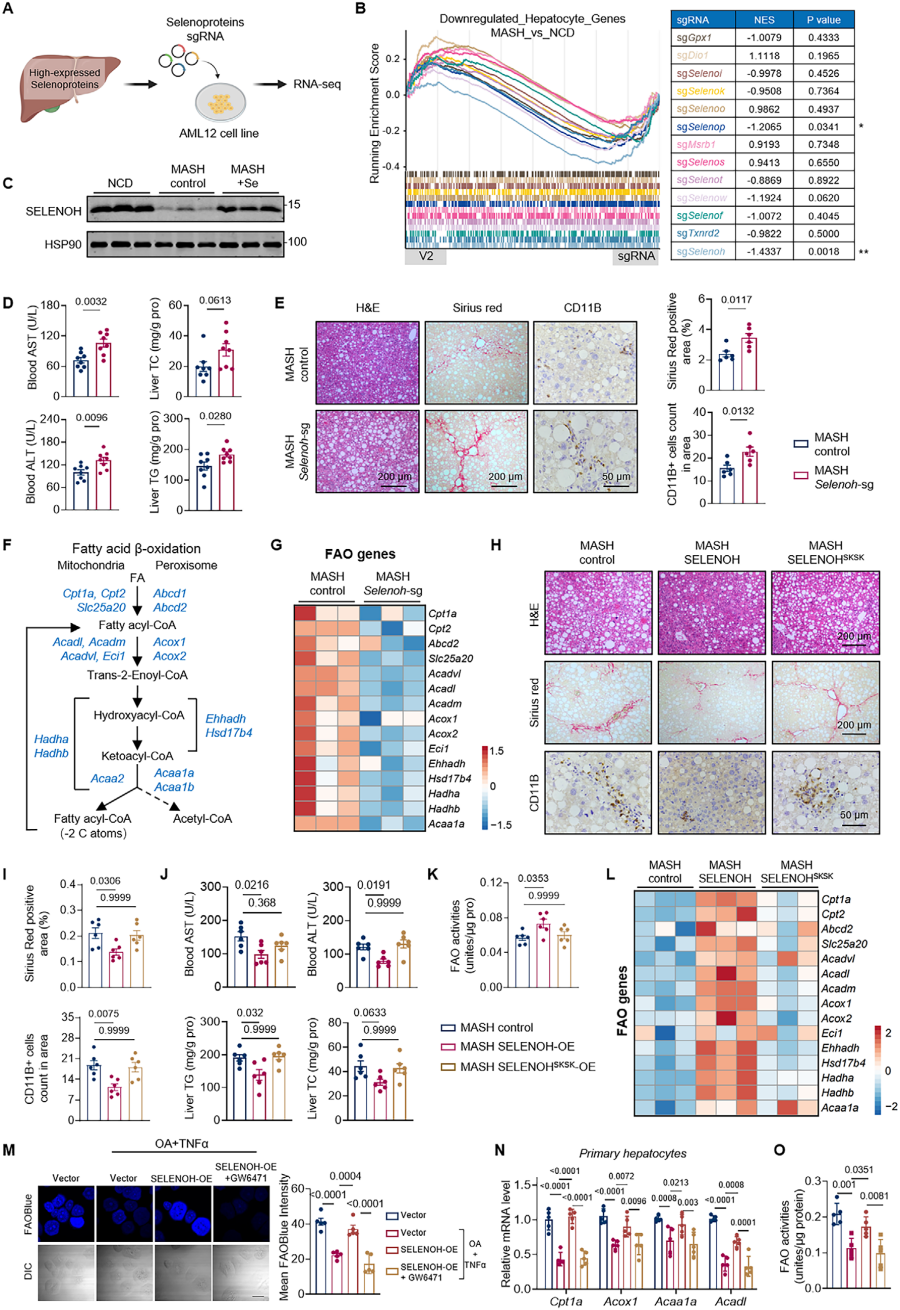
SELENOH regulates fatty acid oxidation and MASH progression. (A) Schematic of the hepatic selenoprotein screening strategy. (B) Multiple GSEA analysis of differentially expressed genes of AML12 in sgRNA group compared to empty lentiviral vectors (lenti‐CRISPR V2). Downregulated genes analyzed in (Figure S4C) are used as the reference gene list. (C) Protein levels of SELENOH in the livers from vehicle and selenium‐treated MASH mice. n = 3 per group. (D) Blood AST and ALT levels, and hepatic TG and TC levels of animals as indicated. n = 6 per group. (E) Representative H&E, Sirius Red and CD11B staining of liver sections from animals as indicated. (F) Schematic of intermediate metabolites and genes involved in each step of fatty acid β‐oxidation (FAO). (G) Heatmap representing the expression of indicated FAO genes in MASH control and MASH *Selenoh*‐sg livers. (H) Representative H&E, Sirius Red and CD11B staining of liver sections from animals as indicated. (I) Percent (%) Sirius Red positive area and CD11B+ cells count based on the immunohistochemistry analysis shown in (H). (J) Blood AST and ALT levels, and hepatic TG and TC levels of animals as indicated. n = 6 per group. (K) FAO activities of liver tissues from animals as indicated. n = 6 per group. (L) Heatmap representing the expression of indicated FAO genes in MASH control, MASH‐SELENOH OE and MASH‐SELENOH^SKSK^ OE livers. (M) Representative images and quantification of FAOBlue intensity in primary hepatocytes from mice injected with AAV‐TBG‐control or AAV‐TBG‐SELENOH. Primary hepatocytes were treated with 10 µm GW6471 (PPARα‐selective antagonist) for 16 h. Cells were stained with FAOBlue (blue) to detect FAO activity. n = 5 independent culture experiments. Scale bars, 50 µm. (N) Relative mRNA levels of indicated FAO genes in primary hepatocytes from mice injected with AAV‐TBG‐control or AAV‐TBG‐SELENOH. Primary hepatocytes were treated with 10 µm GW6471 (PPARα‐selective antagonist) for 16 h. n = 5 independent culture experiments. (O) FAO activities of primary hepatocytes from animals as indicated. n = 5 independent culture experiments. Values are mean ± SEM. The unpaired Student's *t*‐test (D and E) and one‐way ANOVA with post hoc Bonferroni multiple‐comparison test (I–K, M–O) were used for statistical analysis.

Considering our vague understanding in the explicit functions of selenoproteins, we conducted a small‐scale screening, mainly focusing on 13 selenoproteins with relatively high expression in liver (Figure 4A and Table S1). Each of these selenoproteins was individually knocked out via CRISPR Cas9 technology in AML12 cells, followed by RNA sequencing. We speculated that the selenoprotein, whose loss‐of‐function closely recapitulated the transcriptional changes observed in MASH hepatocytes, may possibly regulate MASH development and mediate the response to selenium treatment. By comparing the DEGs upon knockout experiments in AML12 cells and those of hepatocytes in scRNA‐seq data, we identified selenoprotein H (SELENOH) as a potential key mediator whose loss would be most likely to drive the transcriptomic changes in hepatocytes of MASH liver (Figure 4B and S5A). In line with the scRNA‐seq data of MASH livers, genes downregulated in *Selenoh* knockout cells were enriched in fatty acid metabolic processes and steroid metabolic processes (Figure S5B), indicating a dominant disruption of lipid metabolism. As expected, a remarkable reduction in SELENOH protein levels were observed in MASH livers (Figure 4C and S5C), which was markedly recovered upon selenium treatment or SBP2 overexpression (Figure 4C and S5D).

To verify the impact of SELENOH in hepatic lipid metabolism and MASH progression, we then conducted in vivo knockout of *Selenoh* in hepatocytes via AAV vectors and assessed whether *Selenoh* knockout would further exacerbate MASH pathology. The potent effects of MASH diet might mask some effects of SELENOH depletion; we therefore employed a shorter duration (13 weeks) of MASH diet feeding. Compared with control mice, *Selenoh* knockout mice exhibited more severe liver injury and steatosis, as indicated by increased AST and ALT levels, as well as increased hepatic TG and TC contents (Figure 4D). Additionally, liver depletion of SELENOH resulted in more extensive liver fibrosis and inflammation as evidenced by increased Sirius red staining and enhanced infiltration of CD11B‐positive inflammatory cells into liver (Figure 4E). RNA sequencing showed that SELENOH ablation led to downregulation of fatty acid metabolism pathways, with a concomitant enrichment for inflammatory and fibrosis signatures (Figure S5F). More strikingly, genes regulating each step of FAO in both mitochondria and peroxisomes were significantly downregulated in SELENOH‐depleted livers (Figure 4F,G), whereas genes associated with *de novo* lipogenesis (DNL) pathways remained largely unchanged (Figure S5G,H). Further in vitro analysis of primary hepatocytes revealed that SELENOH depletion resulted in significantly decreased FAO gene expression, increased lipid accumulation, as well as reduced basal oxygen consumption rate (OCR) and ATP production (Figure S6A–D).

Conversely, overexpression of SELENOH in hepatocytes via AAV vectors led to obvious improvements in MASH pathology (Figure 4H–J), with significant upregulation of FAO gene expression and FAO activities in liver tissues (Figure 4K,L). In further in vitro studies, primary hepatocytes were treated with oleic acid (OA) and TNFα to mimic the MASH condition, resulting in a significant decrease in FAO activities and the expression of FAO genes (Figure 4M–O). Consistently, SELENOH overexpression (SELENOH‐OE) boosted expression of FAO genes, enhanced FAO activities, and reduced lipid accumulation in primary hepatocytes (Figure 4M–O and S6E,F). Of note, SELENOH predominantly localized to the nucleus with a predicted nuclear localization signal at N terminal [37]. To investigate the function of SELENOH in nucleus, we made a mutant, SELENOH ^SKSK^, to disrupt nuclear localization of SELENOH (Figure S6G). Notably, ectopic expression of SELENOH^SKSK^ failed to attenuate MASH progression, with no significant enhancement of FAO gene expression or FAO activities in liver tissues (Figure 4H–L). Taken together, these findings suggest that SELENOH primarily promotes hepatic expression of FAO genes and hepatic FAO activity, and its transcriptional regulatory function within the nucleus is crucial for mitigating the progression of MASH.

### 2.5 SELENOH Binds to and Activates FAO Gene Transcription in a PPARα‐dependent Manner

**FIGURE 5:**
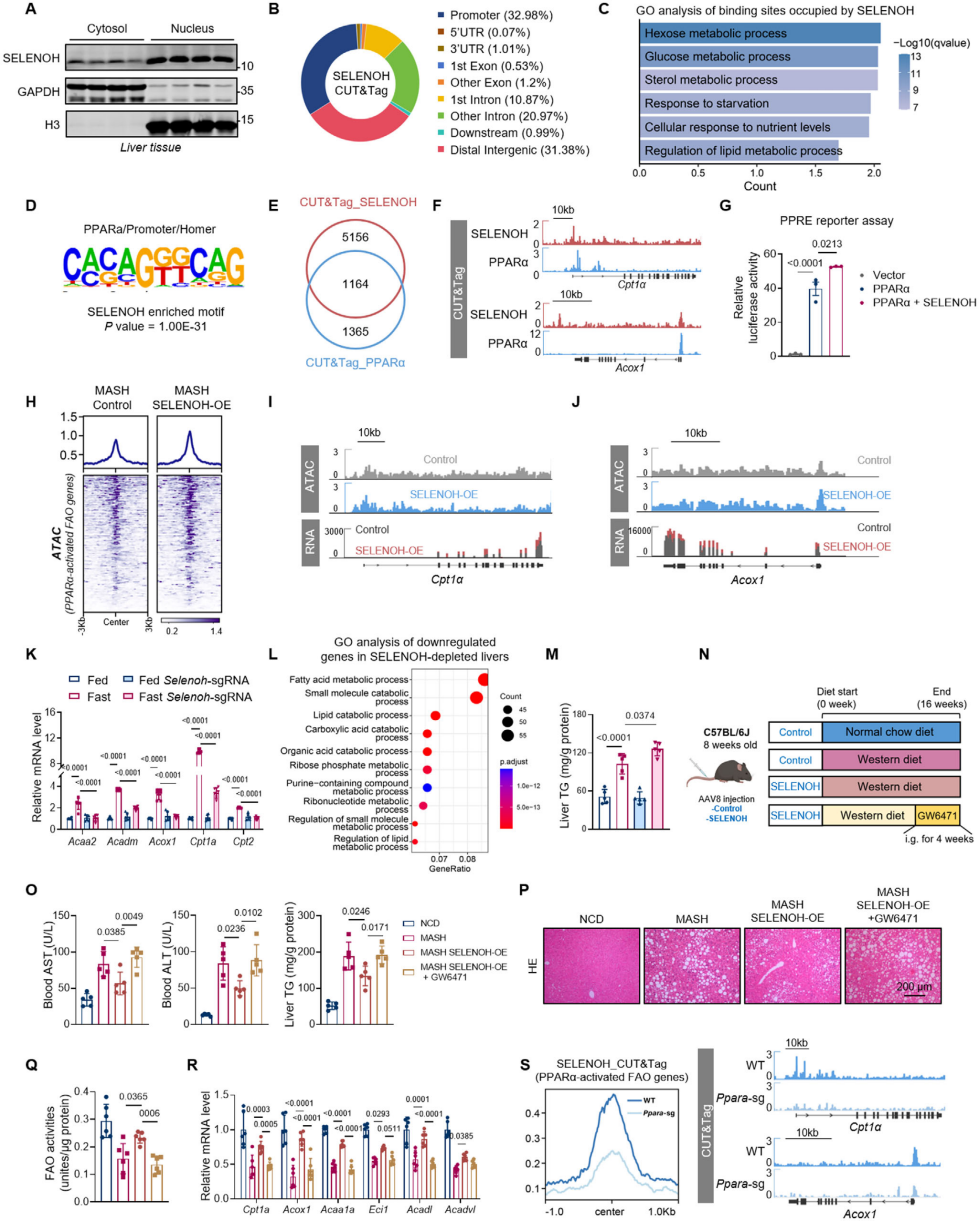
SELENOH binds to and activates fatty acid oxidation gene transcription in a PPARα‐dependent manner. (A) Western blot analysis of liver cytoplasmic and nuclear proteins. (B) Distribution of SELENOH signals in different genomic locations relative to known genes. (C) Barplot showing GO enrichment analysis of binding sites occupied by SELENOH. (D) Motif analysis of SELENOH‐bound sequences in mouse liver. (E) Venn diagram representing the overlapped SELENOH and PPARα peaks detected by CUT&Tag in mouse livers. (F) CUT&Tag tracks of SELENOH and PPARα peaks at the *Cpt1a* and *Acox1* loci. (G) Luciferase reporter experiments using the PPAR response element (PPRE) in HEK293 cells. n = 3 independent culture experiments. (H) Heatmap showing ATAC‐seq signals at PPARα‐activated FAO genes in MASH control and MASH SELENOH‐OE livers. Genes shown in rows were sorted in decreasing order by signal intensity in each condition. (I and J) ATAC‐seq and RNA‐seq tracks of *Cpt1a* and *Acox1* loci. (K) Relative mRNA levels of indicated FAO genes in liver tissues from mice injected with AAV‐TBG‐control or AAV‐TBG‐*Selenoh*‐sgRNA. n = 6 per group. (L) GO enrichment analysis of downregulated genes in SELENOH‐depleted livers. (M) Hepatic TG level of animals as indicated in (K). n = 5 per group. (N) Schematic of the experimental design: GW6471 treatment on SELENOH overexpression mice. (O) Blood AST and ALT levels, and hepatic TG level of animals as indicated. n = 5 per group. (P) Representative H&E staining of liver sections from animals as indicated. (Q) FAO activities of liver tissues from animals as indicated. n = 6 per group. (R) Relative mRNA levels of indicated FAO genes in liver tissues. n = 6 per group. (S) CUT&Tag signals of liver SELENOH at PPARα‐activated FAO genes. CUT&Tag tracks of SELENOH at *Cpt1a* and *Acox1* loci.Values are mean ± SEM. The one‐way ANOVA with post hoc Bonferroni multiple‐comparison test (G, K, M, O, Q, and R) was used for statistical analysis.

Transcriptional regulation of hepatic fatty acid oxidation is largely dependent on some well‐known transcriptional factors, including PPARs and FOXO1 [38]. Given the role of SELENOH in activating hepatic FAO gene expression (Figure 4L) and its nuclear distribution (Figure 5A and S7A), we hypothesized that SELENOH may function in conjunction with classical transcription factors to control the degradation of fatty acids. We carried out CUT&Tag analysis of SELENOH, revealing 32.98% peaks in proximal promoters (Figure 5B). As expected, GO enrichment analysis of binding sites occupied by SELENOH revealed significant enrichment in multiple metabolic pathways including lipid metabolic process (Figure 5C). Transcription factor motif analysis of SELENOH‐bound DNA regions revealed a marked enrichment of PPARα‐binding motif (Figure 5D). Globally, around 46% of the PPARα peaks were overlapped with SELENOH binding sites (Figure 5E). Corresponding with SELENOH's activation of FAO genes, which are typical targets of PPARα, SELENOH and PPARα co‐occupied the promoter regions of FAO genes, including the *Cpt1a* and *Acox1* loci (Figure 5F). In addition, luciferase reporter analysis with PPAR responsive element (PPRE) showed enhanced PPARα activity with SELENOH addition (Figure 5G).

To further confirm the link between SELENOH and PPARα, we first identified a list of FAO genes directly activated by PPARα in mouse livers using available transcriptome datasets (Figure S7B,C). ATAC analysis demonstrated increased chromatin accessibility near these PPARα‐activated FAO genes upon SELENOH overexpression (Figure 5H), aligning with the transcription activation of these genes (Figure 5I,J). The contrary results were also observed upon liver depletion of SELENOH (Figure S7D–F), suggesting the regulatory role of SELENOH in modulating PPARα transcriptional activity.

As an important physiological response, PPARα activity and FAO gene transcription are activated in liver during fasting. Remarkably, hepatocyte‐specific deletion of *Selenoh* significantly abrogated fasting‐induced upregulation of FAO genes in chow‐fed mice (Figure 5K,L and S8A). Indeed, RNA‐seq analysis revealed that around 40% of PPARα‐activated FAO genes were regulated by SELENOH during fasting (Figure S8B). Correspondingly, SELENOH‐depleted mice displayed an increase in hepatic triglycerides upon fasting, probably due to reduced FAO activity (Figure 5M and S8C–G). Similarly, hepatocyte‐specific deletion of *Selenoh* in female mice significantly abrogated the fasting‐induced upregulation of both FAO activity and FAO‐related gene expression, exhibiting elevated hepatic triglyceride accumulation during fasting (Figure S8H–L), demonstrating that SELENOH's regulation of fatty acid oxidation is conserved across sexes. These results altogether place SELENOH as an important regulator in liver FAO under fast conditions.

To figure out whether SELENOH activates the transcription of FAO genes in a PPARα‐dependent manner, we employed the PPARα‐selective antagonist GW6471 for in vivo inhibition. After injection with control or SELENOH overexpression virus, mice were fed a Western diet to induce MASH. During the final month of the modeling period, PPARα was inhibited in the SELENOH‐OE group via administration GW6471 (Figure 5N). As a result, treatment with GW6471 fully abrogated the protective effects of SELENOH in vivo. Specifically, SELENOH‐mediated attenuation of hepatocyte injury and steatosis, upregulation of FAO‐related genes as well as FAO activity enhancement in MASH liver were significantly eliminated by GW6471 treatment (Figure 5O–R).

In further in vitro studies, the effect of SELENOH overexpression on the transactivation of FAO genes, upregulation of FAO activities, and reduction in lipid accumulation in primary hepatocytes was significantly abolished upon *Ppara* knockout (Figure S8M–P), or treatment with GW6471 (Figure 4M,N). Furthermore, CUT&TAG analysis revealed overall binding of SELENOH on PPARα‐activated FAO genes was significantly decreased upon PPARα depletion (Figure 5S). Collectively, these results indicate that SELENOH works as a positive regulator of FAO gene transactivation in a PPARα‐dependent manner.

### 2.6 SELENOH Interacts With Activated PPARα and Facilitates Its Recruitment of Coactivator P300

**FIGURE 6:**
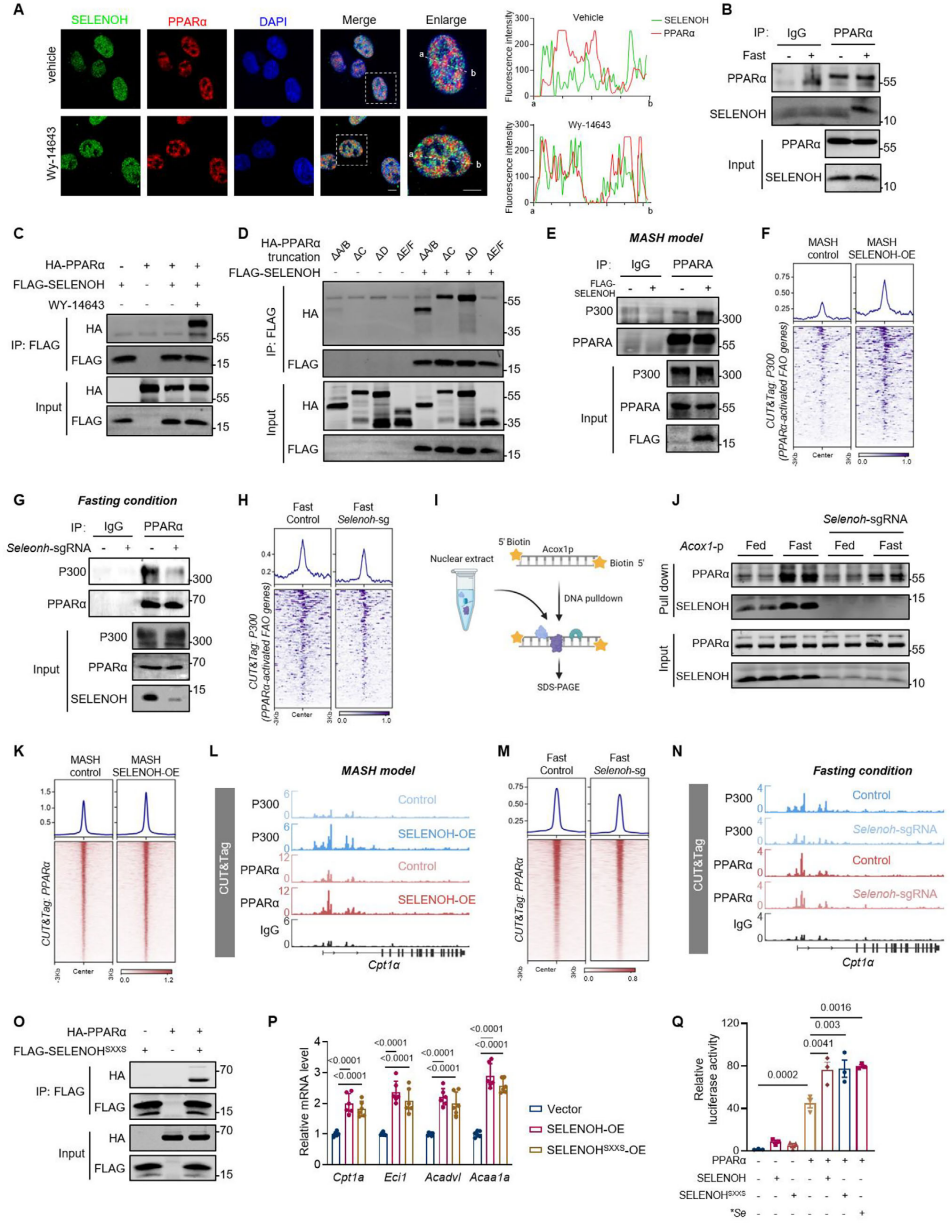
SELENOH interacts with activated PPARα and facilitates the genomic recruitment of PPARα‐P300 complex. (A) Representative immunofluorescence of SELENOH and PPARα in U2OS, Scale bars, 5 µm. (B) Endogenous co‐immunoprecipitation (co‐IP) analysis between PPARα and SELENOH in nuclear proteins from mouse livers under fasting or fed conditions. (C) Co‐IP of HA‐PPARα and FLAG‐SELENOH in HEK293T cells treated with vehicle or wy‐14643. (D) Co‐IP of different PPARα truncations with FLAG‐SELENOH in HEK293T cells treated with wy‐14643. (E) Endogenous co‐IP of PPARα and P300 in liver nuclear proteins from MASH control and MASH SELENOH‐OE mice. (F) Heatmap showing CUT&Tag signals of P300 at PPARα‐activated FAO genes in MASH control and MASH SELENOH‐OE livers. (G) Endogenous co‐IP of PPARα and P300 in liver nuclear proteins from mice injected with AAV‐TBG‐control or AAV‐TBG‐*Selenoh*‐sgRNA under the fasting condition. (H) Heatmap showing CUT&Tag signals of P300 at PPARα‐activated FAO genes in livers from mice injected with AAV‐TBG‐control or AAV‐TBG‐*Selenoh*‐sgRNA under the fasting condition. (I) Schematic of DNA pulldown assay. (J) Western blot analysis of biotin‐*Acox1* promoter (*Acox1*‐p) pull‐down of nuclear protein extracts from indicated liver tissues. (K) Heatmap showing CUT&Tag signals of PPARα in MASH control and MASH SELENOH‐OE livers. (L) CUT&Tag tracks of P300 and PPARα peaks at the *Cpt1a* locus. (M) Heatmap showing CUT&Tag signals of PPARα in livers from mice injected with AAV‐TBG‐control or AAV‐TBG‐*Selenoh*‐sgRNA under the fasting condition. (N) CUT&Tag tracks of P300 and PPARα peaks at the *Cpt1a* locus. (O) Co‐IP of HA‐PPARα and FLAG‐SELENOH^SXXS^ in HEK293T cells treated with vehicle or wy‐14643. (P) Relative mRNA levels of indicated FAO genes in primary hepatocytes from mice injected with AAV‐TBG‐control, AAV‐TBG‐SELENOH, or AAV‐TBG‐SELENOH^SXXS^. n = 6. (Q) Luciferase reporter experiments using the PPAR response element (PPRE) in HEK293T cells. Cells were transfected with indicated plasmid with or without 1 µM selenium (Se) treatment. n = 3 independent culture experiments.Values are mean ± SEM. The one‐way ANOVA with post hoc Bonferroni multiple‐comparison test (P and Q) was used for statistical analysis.

PPARα controls the transcription of genes involved in the FAO process, which may be influenced by its protein stability in the nucleus, the ability of ligand binding, and the presence of various transcriptional coactivators [39, 40]. Depletion or overexpression of SELENOH did not influence the protein level of PPARα in nucleus (Figure S9A,B). Notably, activation of PPARα by wy‐14643 (a synthetic PPARα agonist) induced the co‐localization of endogenous SELENOH and PPARα (Figure 6A). Co‐immunoprecipitation (co‐IP) analysis demonstrated that SELENOH specifically bound to activated PPARα in liver tissue from fasted mice, or in cells treated with wy‐14643 (Figure 6B,C). Subsequent domain mapping revealed that the E/F domain of PPARα, representing its ligand‐banding domain (LBD), was crucial for mediating the interaction with SELENOH (Figure 6D and S9C). Molecular docking analyses consistently showed that SELENOH binds with LBD of PPARα by hydrogen bonds and salt bridges (Figure S9D). Ligand binding stabilizes the PPARα‐RXRα heterodimer, enhancing PPARα transcriptional activity [41]. However, SELENOH depletion did not affect the dimerization of PPARα and RXRα (Figure S9E). On the other hand, following ligand binding, the LBD of PPARα undergoes conformational changes, thereby facilitating the recruitment of co‐activators, such as CBP/p300 and SRC‐1 to regulate the transcription levels of target genes [42, 43]. We found SELENOH overexpression significantly enhanced the interaction between PPARα and P300 (Figure 6E). CUT&Tag assay further indicated that SELENOH facilitated the recruitment of P300 to PPARα‐activated FAO genes (Figure 6F). Correspondingly, liver SELENOH depletion decreased the interaction of PPARα and P300 and reduced the recruitment of P300 to PPARα‐activated FAO genes under fasting condition (Figure 6G,H).

We next examined whether SELENOH further affects the genomic binding affinity of PPARα. Using a biotin‐labeled *Acox1* promoter sequence as a bait for DNA pull‐down assays (Figure 6I), we observed enhanced binding of PPARα and SELENOH to the DNA probe in liver tissue from fasted animals (Figure 6J). Notably, SELENOH depletion significantly impaired the DNA binding affinity of PPARα in liver tissues during fasting (Figure 6J). Further CUT&Tag analysis of PPARα revealed enhanced genome binding affinity of PPARα upon SELENOH overexpression in MASH livers (Figure 6K,L and S9F), while SELENOH depletion significantly impaired the genomic binding affinity of PPARα in fasted liver tissues (Figure 6M,N and S9G). Recruitment of P300 can create an open chromatin conformation, which might eventually lead to further enhanced genome‐binding ability of PPARα and transactivation of FAO genes. These results underscore the essential role of SELENOH‐PPARα interaction in recruiting the coactivator P300, which in turn influences genomic binding affinity and transcriptional activity of PPARα.

To further clarify the role of SELENOH within this PPARα/P300/SELENOH transactivation complex, we focused on its previously reported antioxidant enzyme function [44]. Mutation of catalytic cysteine and selenocysteine residues in the redox‐related CXXU motif to serine significantly reduce the enzyme activity of SELENOH [44]. Intriguingly, SELENOH mutant (SELENOH^SXXS^) did not affect its interaction with PPARα (Figure 6O), while maintaining the ability to activate FAO gene expression (Figure 6P), to reduce lipid accumulation (Figure S9H), and to enhance PPARα transcriptional activity (Figure 6Q). These results indicate that SELENOH enhances FAO gene expression independent of its redox activity, more likely serving as an adapter protein for the assembly of PPARα transactivation complex.

### 2.7 Selenium Supplementation Attenuates MASH Progression via the SELENOH‐PPARα Axis

**FIGURE 7:**
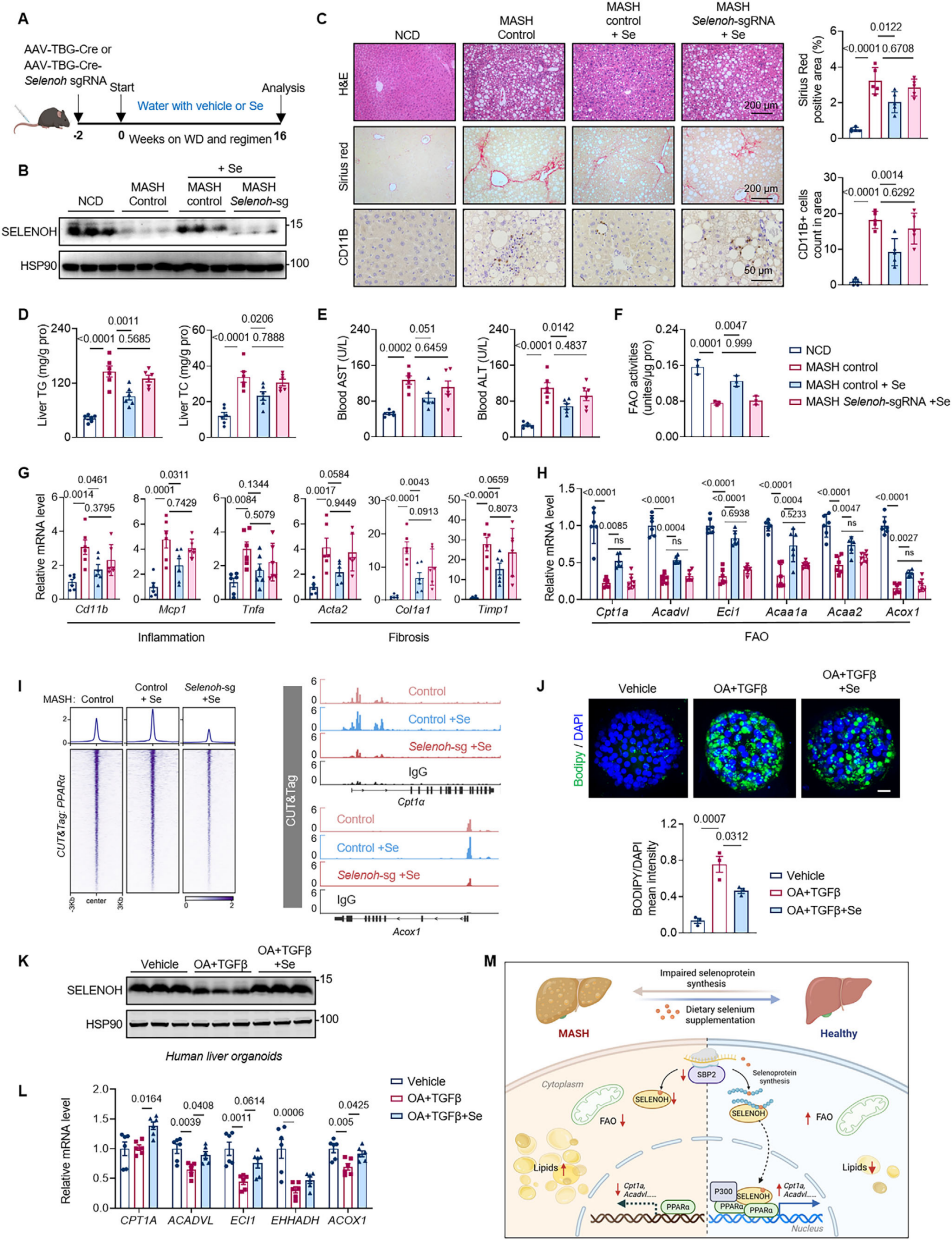
Selenium supplementation attenuates MASH progression via the SELENOH‐PPARα axis. (A) Schematic of the experimental design: selenium treatment on *Selenoh* knockout mice. (B) Protein levels of SELENOH in the livers from animals as indicated. n = 3 per group. (C) Representative H&E, Sirius Red and CD11B staining of liver sections from animals as indicated. (D) Hepatic TG and TC levels of animals as indicated. n = 6 per group. (E) Blood AST and ALT levels of animals as indicated. n = 6 per group. (F) FAO activities of liver tissues from animals as indicated. n = 3 per group. (G) Relative mRNA levels of genes related to hepatic inflammation and fibrosis. n = 6 per group. (H) Relative mRNA levels of indicated FAO genes in liver tissues from animals as indicated. n = 6 per group. (I) Heatmap showing CUT&Tag signals of PPARα in MASH control, MASH + Se and MASH *Selenoh*‐sg +Se livers. (J) Representative images of lipid accumulation in Human liver organoids (HLOs). HLOs were stained with BODIPY for fat accumulation (green) and DAPI for the nucleus (blue). Scale bars, 20 µm. (K) Protein levels of SELENOH in HLOs after indicated treatments. (L) Relative mRNA levels of indicated FAO genes in HLOs after indicated treatments. n = 6 independent culture experiments (M) Schematic illustration of selenium in regulating MASH progression. In healthy livers, when PPARα is activated, SELENOH interacts with PPARα, which stabilizes the PPARα‐P300 complex and promotes its chromatin recruitment for fatty acid oxidation (FAO) gene activation. In contrast, livers with MASH exhibits attenuated selenoprotein translation due to reduced SBP2 expression—a key regulator of selenoprotein biosynthesis. This impairment lowers hepatic SELENOH levels, compromising PPARα genomic recruitment and dampening FAO gene transcription. Restoring SBP2 expression or supplementing dietary selenium can significantly recover selenoprotein production, thereby enhancing FAO and mitigating MASH progression. Values are mean ± SEM. The one‐way ANOVA with post hoc Bonferroni multiple‐comparison test (C–H, J, and L) was used for statistical analysis.

We identified the liver selenoprotein SELENOH as the key regulator of PPARα‐mediated fatty acid oxidation, with its expression could be restored by selenium supplementation. To verify the possible beneficial role of SELENOH in the context of selenium treatment for MASH, we administered selenium supplementation to control and *Selenoh* knockout mice (Figure 7A). After MASH modeling, in comparison with the improvement on control mice, selenium intervention failed to improve MASH phenotypes in *Selenoh* knockout mice, as evaluated by histological features and biochemical analysis (Figure 7B–G). Notably, selenium treatment had no impact on the expression of FAO genes and FAO activities in *Selenoh* knockout mice (Figure 7F,H). In addition, the genomic recruitment of PPARα to its target genes was restored following selenium supplementation, a process markedly impaired in the absence of SELENOH (Figure 7I). These results underscored selenium supplementation attenuated MASH progression via the SELENOH‐PPARα axis.

To further corroborate the relevance of these findings to humans, we generated human liver organoids (HLOs) from human pluripotent stem cells and subjected them to TGFβ and OA treatment to replicate MASH progression [45, 46]. Significantly, we observed that selenium supplementation in the medium substantially mitigated lipid accumulation in HLOs (Figure 7J). This supplementation restored the expression of SELENOH and enhanced the expression of FAO genes in the organoids (Figure 7K,L). These results underscore the critical role of SELENOH in mediating the beneficial effects of selenium supplementation, particularly in the context of FAO gene expression and activity. The findings from human organoids further support the potential therapeutic relevance of selenium supplementation in combating MASH progression.

## 3 Discussion

Selenium, a vital trace element, is known to play a crucial role in various physiological processes in the body. In this study, we found a notable decrease in serum selenium levels of human subjects with liver steatosis and MASH. Selenoprotein P (SELENOP), a major format of serum selenium, is mainly secreted by liver. Decreased serum SELENOP levels in patients with hepatic steatosis indicate hepatic selenium insufficiency, which is further validated in liver samples from MASH patients. Importantly, serum selenium level is identified as a significant protective factor against MASH development in NHANES database. Consistent with the human studies, our murine MASH models recapitulate the low‐selenium status in livers, manifested by significantly reduced selenoprotein synthesis due to diminished SBP2 expression. Moreover, reinstating SBP2 expression or introducing dietary selenium supplementation markedly restore selenoprotein synthesis and alleviate MASH progression. Mechanistically, selenium supplementation enhanced the transcription of genes involved in fatty acid oxidation through selenoprotein H (SELENOH)‐mediated activation of the PPARα signaling pathway. Specifically, SELENOH predominantly interacts with the ligand‐binding state of PPARα, facilitating P300 recruitment and thereby enhancing the genomic recruitment and transcriptional activity of PPARα (Figure 7M). These findings elucidate the role of the selenium‐SELENOH‐PPARα axis in regulating liver lipid homeostasis, underscoring the pivotal functions of selenium and SELENOH in MASH development. It is worth noting that a study was published during our submission period which also reports the beneficial effects of biomimetic selenium‐nanocomposites in treating MASH [47]. Although this study does not provide extensive mechanistic detail, it serves as an independent validation of selenium's therapeutic potential for this condition.

Selenium insufficiency has been observed in various chronic liver diseases, similar to the decreased hepatic selenium and selenoprotein levels we observed in MASH. This led us to explore the causes of selenoprotein downregulation. Dietary selenium comes in two forms: inorganic selenium (mainly sodium selenite), which is absorbed by diffusion and reduced by TXNRD1 to selenide for direct selenoprotein synthesis; and organic selenium (primarily selenomethionine), which enters via amino acid transporters and is mobilized for selenoprotein synthesis through selenium recycling (transsulfuration and selenocysteine decomposition) (Figure S10A) [48]. Our mouse models showed no significant differences in the aforementioned selenium metabolism proteins (Figure S10B,C). Notably, experimental animal feed is typically supplemented with inorganic selenium, whereas human intake relies primarily on organic selenium sources [2, 49]. Consequently, further investigation is warranted to determine whether recycling of organic selenium influences MASH progression in humans. Selenoprotein synthesis is highly regulated. Here, we found impaired selenoprotein translation during MASH progression, largely due to reduced levels of SBP2—a key translational regulator—contributing to functional selenium insufficiency in the MASH liver.

Under conditions of selenium restriction, there is a hierarchy of selenoprotein expression in cells, with certain housekeeping selenoproteins (e.g., GPX4) synthesized at the expense of stress‐related selenoproteins (e.g., GPX1) [3, 50]. This pattern aligns with our observations in MASH livers, demonstrating a pronounced decline in stress‐induced selenoproteins, such as GPX1, MSRB1 and SELENOH, along with stable expression of housekeeping selenoproteins such as GPX4 and TXNRD1. The hierarchy  of differential selenoprotein expression is regulated by selenium availability, mainly at the step of Sec incorporation [51, 52, 53]. Decoding the UGA stop codon as selenocysteine (Sec) is the rate‐limiting step in selenoprotein biosynthesis. This process relies on a Sec‐insertion sequence (SECIS) in the selenoprotein mRNA and special trans‐acting protein factors (such as SBP2 and eEFSec) [29]. This system enables the UGA/Sec recoding while preventing premature translation termination [54, 55]. Notably, SBP2 specifically binds the SECIS element and serves as the scaffold to mediate co‐translational Sec incorporation at UGA codons [56]. Previous studies have demonstrated that differential binding affinities of SBP2 for various SECIS elements may determine the hierarchy pattern of selenoprotein expression [57, 58]. Consistent with these findings, our data demonstrated that reduced SBP2 expression resulted in reduced levels of stress‐related selenoproteins. However, whether the SBP2‐SECIS interactions are perturbed in MASH liver requires further investigation.

Selenium supplementation increases the efficiency of Sec incorporation and selenoprotein abundance, thereby improving MASH phenotypes. Usually, selenium serves as an essential component of multiple critical antioxidant enzymes (selenoproteins) to attenuate the development of diseases, especially metabolic diseases. However, clinical trials investigating antioxidant monotherapy (e.g., vitamin E) for MASH failed to meet primary endpoints due to insufficient efficacy in attenuating hepatic fibrosis [59, 60]. Additionally, GPX4 protein levels were unaffected by either MASH progression or selenium supplementation, indicating that GPX4‐mediated ferroptosis may not be involved in the effect of selenium on MASH. Our single‐cell RNA sequencing data revealed that selenium supplementation significantly reversed hepatic lipid metabolic pathways, prompting us to investigate the potentials of selenoproteins in directly regulating liver lipid metabolism. A small‐scale selenoprotein screening identified that *Selenoh* knockout introduced significant alterations in lipid metabolism‐related gene expression. In vivo studies validated the critical role of SELENOH in MASH progression and suggested it as a primary effector of selenium intervention. Previous experimental studies have reported that SELENOH primarily localized in nucleus, with potential to regulate gene expressions, particularly those implicated in *de novo* glutathione synthesis and phase II detoxification in response to redox status in HT22 cells [37]. Nevertheless, the precise biological function of SELENOH, particularly within an in vivo setting, remains enigmatic.

Our study shed light on the role of SELENOH in regulating FAO gene expression. SELENOH functions as a scaffold that interacts with ligand‐bound PPARα, facilitating its recruitment of P300, and subsequent transcriptional activation of target genes. Notably, the interaction between PPARα and SELENOH occurs in a ligand‐dependent manner, indicating the essential function of SELENOH in modulating PPARα activation under physiological conditions. Liver‐specific *Selenoh* knockout markedly suppressed expression of FAO genes in hepatocytes, particularly during fasting‐induced PPARα activation. Furthermore, in the pathological state of MASH, impaired selenoprotein synthesis results in reduced SELENOH expression, which subsequently diminishes FAO activity and promotes MASH progression. Intriguingly, catalytic center mutations in SELENOH did not alter its regulatory effects on FAO gene expression. While selenoproteins typically exert their biological effects via their enzymatic activities, our finding highlights an enzyme‐independent mechanism of SELENOH in epigenetically modulating transcription of genes involved in liver lipid metabolism. Though our study primarily delves into the regulatory role of SELENOH in liver lipid metabolism, our CUT&Tag seq and RNA seq analyses implicate that SELENOH also directly targets genes beyond FAO, including those involved in hexose metabolic processes and monosaccharide metabolic processes. The mechanisms through which SELENOH regulates these pathways, thereby governing broader liver functions beyond lipid metabolism, warrant further investigation.

PPARα, a ligand‐activated transcription factor, functions as a master regulator of lipid metabolic gene networks [40]. Several drugs targeting PPARα are under clinical study for MASH treatment [18, 61, 62]. Beyond its canonical transactivation function, PPARα exerts ligand‐dependent transrepressive effects on pro‐inflammatory signaling pathways through protein‐protein interactions [63, 64]. Our study identified the therapeutic potential of the SELENOH‐PPARα axis in ameliorating inflammation and fibrosis in MASH, potentially through the beneficial effects of SELENOH on hepatic lipid metabolism. However, whether SELENOH directly enhances PPARα‐mediated suppression of inflammatory signaling pathways requires further investigation.

As noted above, our findings underscore the substantial beneficial impact of selenium on MASH and identify SELENOH as a critical contributor via functional screening of selenoproteins in hepatocytes. Other selenoproteins, in addition to SELENOH, may also modulate liver function and influence MASH development [35, 65, 66]. In our screening of hepatic selenoproteins, the deficiency of SELENOH most closely recapitulates the transcriptional profile in MASH hepatocytes, while cells with sgRNAs targeting *Selenop* and *Selenow* also exhibits comparable alterations that warrant further investigation. Furthermore, selenium supplementation also causes functional disturbances in other cell types beyond hepatocytes. Our scRNA‐seq analysis revealed that selenium treatment significantly alters the lipid associated macrophage signature in immune cells. However, its effects on the complex liver niches, including immune cells, endothelial cells, and stellate cells, require further study.

When proposing selenium supplementation as a novel therapeutic strategy for MASH, two factors are critical to consider: sex differences and baseline selenium levels. First, sex‐specific aspects, including genetic differences and sex hormones, modulate selenium metabolism and selenoproteins [67, 68], which may underlie the sex‐specific effects of selenium on MASH. When the analysis of NHANES data was stratified by sex, multivariable logistic regression indicated that serum selenium was an independent protective factor against MASH in males, but not in females (Figure S11). Supporting our observation, two large‐scale studies also reported marked sexual dimorphism in selenium status with regard to metabolic syndrome [69, 70]. This pronounced sexual dimorphism may stem from the regulation of selenium metabolism by sex hormones at multiple levels—including selenium absorption, selenomethionine metabolism, and subsequent Sec availability for selenoprotein synthesis [67]. Given the complexity of the underlying mechanisms, this area merits dedicated investigation in future studies.

Second, the health effects of selenium exhibit an inextricable U‐shaped relationship with baseline selenium status, indicating a narrow beneficial window [2]. While selenium supplementation can benefit individuals with low status, elevated plasma levels (>150 µg/L) are associated with increased mortality risk [71, 72]. Therefore, selenium supplements are advisable only for those with a deficiency and may be harmful for individuals with adequate‐to‐high baseline status.

Collectively, the potential effects of selenium supplementation to MASH patients warrants more careful study.

## 4 Conclusion

In summary, our study unveils a specific pathophysiological mechanism through which selenium and selenoprotein H are involved in MASH development. We demonstrate that SELENOH is a key regulator of PPARα‐mediated gene transcription and highlight the therapeutic potential of selenium supplementation in treating MASH.

## 5 Experimental Section

### 5.1 Animals

All animal experiments in this study were performed according to protocols approved by the Institutional Animal Care and Use Committee of the Shanghai Institute for Nutrition and Health (ethical committee approval no.SINH‐2022‐DQR‐1). Wild‐type C57BL/6J mice were purchased from Shanghai SLAC Laboratory Animal Co.,Ltd. All mice were housed in a specific pathogen‐free (SPF) facility at the temperature of 22°C–26°C, with access to food and water ad libitum, under a 12‐h light/dark cycle. All animals presented a healthy status and male mice were used for all experiments.

For liver‐specific overexpression of *Selenoh* or *Secisbp2*, male C57BL/6J mice (6–8 weeks old) were administrated with adeno‐associated virus 8 (AAV8) vectors expressing the gene under a liver‐specific TBG promoter via tail vein injection (2 × 10^12^ vector genomes per mouse). AAV8 vectors expressing luciferase were used as control viruses.

For liver‐specific depletion of *Selenoh* or *Ppara*, 8‐week‐old male heterozygous Cas9 knockin mice (RosaCas9^+/−^) were administrated with AAV8 vectors expressing Cre recombinase and sgRNAs targeting individual genes via tail vein injection (2 × 10^11^ vector genomes per mouse). The sequences of sgRNAs used in this study are listed in Table S2. AAV8 vectors with Cre recombinase and luciferase cassettes and no sgRNA were used as control viruses.

For the establishment of the western diet (WD) ‐induced murine MASH model, mice were subjected to a 16‐week regimen, consisting of WD (42 kcal% Fat, 42 kcal% Carbohydrate and 1.25% Cholesterol, 0.16 ppm selenium, Teklad diets, TD.120528) and a high sugar solution (23.1 g/L D‐fructose and 18.9 g/L D‐glucose). In addition, mice were injected intraperitoneally with CCl_4_ (0.2 mL/kg body weight, 1:5 diluted in corn oil) once per week.

For methionine‐ and choline‐deficient (MCD) diet‐induced murine MASH model, mice were fed a MCD diet (Research Diets, A02082002B) for 6 weeks.

For gubra‐Amylin NASH (GAN) diet‐induced murine MASH model, mice were fed with a GAN diet (40 kcal% Fat, 20 kcal% Fructose and 2% Cholesterol, 0.23 ppm selenium, Xietong Shengwu, XT310) for 24 weeks. To further induce MASH resolution, mice were then switched from GAN diet to a normal diet (Shanghai Laboratory Animal Center, P1103F). The indicated mice were fed a normal diet for 2–8 weeks as indicated.

### 5.2 Clinical Cohort 1

All experiments involving patients were approved by the Science and Technology Ethics Committee of Linyi People's Hospital in accordance with the principles of the Declaration of Helsinki (202501‐H‐005). All the biospecimens of human participants of clinical cohort 1 were obtained from the Linyi People's Hospital. Written informed consent was obtained from all human participants prior to the research. Clinical cohort 1 included 30 participants, categorized into healthy group (n = 15) and hepatic steatosis group (n = 15) according to the hepatic steatosis index (HSI), which is a simple, efficient screening tool reflecting steatotic liver diseases. At values of <30.0 or >36.0, HSI is able to rule out MASLD or detect MASLD with high sensitivity [26]. Variables including body mass index (BMI), ALT/AST ratio, sex and diabetes mellitus were used to devise HSI. The clinical characteristics of the 30 subjects are listed in Table S3.

### 5.3 Clinical Cohort 2

Data in cohort 2 were collected from the National Health and Nutrition Examination Survey (NHANES) 2017‐March 2020 cycle, which included liver ultrasound transient elastography data. For this study, we included adult participants (age 18–65 years) with complete medical examination records and excluded participants with excessive alcohol consumption (≥ 30 and 20 g/day in men and in women, respectively) and viral hepatitis. MASLD and MASH were defined following several criteria reported by recent epidemiological studies [73, 74, 75]. Briefly, MASLD (≥5% steatosis) were defined as controlled attenuation parameter (CAP) scores of ≥ 302 dB/m. Liver fibrosis was determined by liver stiffness measurements (LSM) value. MASLD participants with LSM ≥ 8.2 kPa were considered as MASH patients. Participants with HSI < 30, CAP < 302 and LSM < 8.2 were defined as non‐MASLD. Individuals with high LSM values (> 13.6) without MASLD signature (CAP < 302) were defined as cryptogenic cirrhosis and removed from the analysis. Finally, 1304 participants were used to analyze the association between serum selenium level and MASLD/MASH.

### 5.4 Cell lines

The murine AML12 hepatocytes, human osteosarcoma U2OS and human embryonic kidney 293T (HEK293T) cells (Cell Bank, Type Culture Collection Committee, Chinese Academy of Sciences) were grown in monolayer at 37°C in 5% CO2. HEK 293T and U2OS cell lines were maintained in DMEM (Gibco, C11995500CP) containing 10% fetal bovine serum [FBS, (Gibco, 16000044)] and 1% penicillin/streptomycin (PS). AML12 cell lines were maintained in DMEF‐12 (Gibco, C11330500CP) containing 10% FBS, 1% ITS Liquid Media Supplement (Sigma, I3146), and 40 ng/mL dexamethasone (Sigma, D4902).

### 5.5 Plasmids

The *Secisbp2* (NM_029279.2), *Selenoh* (NM_001033166.3) and *Ppara* (NM_001113418.1) gene constructs were amplified from mouse liver sample, and then cloned into pCDH‐EF1‐MCS‐T2A‐PuroR plasmid (CD520A‐1, System Biosciences). The mutated construction of SELENOH^SXXS^ and SELENOH^SKSK^ and different PPARα truncations were developed by the standard PCR protocol, and the oligo sequences can be found in Table S4. For plasmids used in AAV packaging, the liver‐specific CRISPR AAV vector, AAV‐TBG‐Cre‐luciferase‐sgRNA, used in this study was constructed by modifying the pAAV‐GFP plasmid (Cell Biolabs). The original CMV promoter was replaced with the liver‐specific TBG promoter, and the original GFP cassette was replaced with the Cre‐T2A‐luciferase‐sgRNA cassette for expression of Cre recombinase, luciferase and sgRNA, in which the sgRNA scaffold was derived from the lentiCRISPR‐V2 plasmid (Addgene, 52961). This vector was used to insert sgRNA sequences targeting specific genes or used directly as a control vector. The liver‐specific AAV vector used for overexpression was constructed by modifying AAV‐TBG‐Cre‐luciferase‐sgRNA plasmid, with the Cre‐luciferase‐sgRNA cassette replaced with the coding DNA sequence of the target protein. AAV packaging was following a detailed protocol as described previously [76].

### 5.6 SgRNA Screening in AML12 Cells

The sgRNAs designed to target a specific gene were generated using the online tool at http://chopchop.cbu.uib.no/ or https://tools.synthego.com/#/, and then inserted into lentiCRISPR‐V2 plasmid (Addgene, 52961). Lentiviruses were produced in HEK293T cells by transfection of packaging plasmids pMDLg/pRRE (Addgene, 12251), pRSV‐Rev (Addgene, 12253) and pMD2.G (Addgene, 12259) together with individual lentiviral sgRNA vectors. The AML12 cells were infected with lentiviral particles carrying different sgRNAs. After 72 h of infection, the culture media was changed to fresh puromycin‐containing media to select for infected cells. Cells were collected, and the whole cell lysates were subjected to SURVEYOR assay to confirm genomic cleavage or western blotting assay to examine the level of the targeted protein. Cells with successful targeting of individual protein were subjected to following analysis.

### 5.7 Cytoplasmic and Nuclear Protein Extraction

Cytoplasmic and nuclear proteins were isolated from liver tissue using subcellular protein fraction kit (87790, ThermoFisher). Briefly, approximately 50 mg of liver tissue was homogenized in 500 µL Cytoplasmic extraction buffer plus protease inhibitors cocktail using the Dounce tissue homogenizer. The tissue homogenate was then centrifuged at 500 g for 5 min, and the supernatant was cytoplasmic protein lysate. The membrane extraction buffer containing protease inhibitors was then added to the pellet and incubated at 4°C for 10 min. Samples were then centrifuged at 3000 g for 5 min to separate the membrane extract. Finally, the nuclear pellet was lysed in nuclear extraction buffer with CaCl_2_ and micrococcal nuclease at room temperature for 15 min and centrifuged at 12 000 g for 15 min. The resulting supernatant, containing the nuclear protein lysate, was collected for subsequent western blotting assay, immunoprecipitation assay, or DNA pull‐down assay.

### 5.8 Western Analysis

Cell and tissue lysates were subjected to electrophoresis through SDS‐PAGE and transferred to nitrocellulose or polyvinylidene difluoride (PVDF) membranes, and then blotted with antibodies. The primary antibodies used in this study were as following: GPX1 (A23519, ABclonal), MSRB1 (15333‐1‐AP, Proteintech), SELENOH (Ab151023, Abcam), SBP2 (Ab210791, Abcam), GPX4(A11243, ABclonal), TXNRD1(A4725, ABclonal), EFSEC(10628‐1‐AP, Proteintech), HSP90 (13171‐1‐AP, Proteintech), PPARα (Ab227074, Abcam), RXRA(21218‐1‐AP, Proteintech), SELENOP (GB111081, Servicebio), EP300 (57625S, Cell signaling), GAPDH (10494‐1‐AP, Proteintech), Histone H3 (12648S, Cell signaling), DYKDDDDK tag (20543‐1‐AP, Proteintech), HA (66006‐2‐IG, Proteintech), αSMA (GB111364, Servicebio). The secondary antibodies used in this study were IRDye 680RD Donkey anti‐Mouse IgG antibody (926‐68072, LI‐COR), IRDye 800CW Donkey anti‐Rabbit IgG antibody (926‐32213, LI‐COR). A Li‐COR Odyssey system (Li‐COR Biosciences) and Image Studio Lite Ver 5.2 were used for fluorescent immunoblotting signal collection, and signal quantification.

### 5.9 Co‐Immunoprecipitation

To analyze the interaction between FLAG‐SELENOH and different HA‐PPARα truncations, HEK293T cells were transfected with tagged proteins, and then lysed in RIPA buffer (Millipore, 20188). The lysates were incubated with anti‐FLAG M2 Magnetic Beads (Sigma, M8823) at 4°C overnight, followed by western analysis. To analyze the interaction between endogenous PPARα with SELENOH, nuclear proteins were isolated from liver tissue using subcellular protein fraction kit. The isolated nuclear proteins were incubated with PPARα antibody for 1 h at 4°C, followed by the addition of Protein A/G PLUS‐Agarose (Santa Cruz, sc‐2003). Samples were then incubated overnight at 4°C with gentle agitation. The beads were later washed three times with lysis buffer before western analysis. Normal rabbit IgG (Cell Signaling, 2729S) was used as a negative control.

### 5.10 DNA Pull‐Down Assay

The detection of DNA‐binding proteins was performed using a promoter pull‐down assay procedure as described previously [77]. In brief, we amplified the PPARα‐binding DNA region of *Acox1* (Acox1‐p) from mouse genomic DNA using primers labeled with 5’‐biotin: *Acox1*‐p forward, CTGCAATCCCCGACGCT;*Acox1*‐p reverse, ACTGGTGAAAACTGGCTGTCT. The biotinylated *Acox1*‐p probes were immobilized on Streptavidin Magnetic Beads (HY‐K0208, MCE) in DNA binding buffer (5 mmol/L Tris pH 7.5, 0.5 mmol/L EDTA, 1 mol/L NaCl). Nuclear proteins were isolated from liver tissue, and 0.5 mg of nuclear extract was incubated with the probe‐bound beads at 4°C overnight. To reduce non‐specific DNA‐binding protein interactions, calf thymus DNA was added during the incubation. After incubation, the supernatant was discarded, and the beads were washed twice with lysis buffer and three times with PBS. The beads were then resuspended in 50 µL loading buffer, boiled for 5 min, and were then separated by SDS‐PAGE for later western analysis.

### 5.11 Polysome Fractionation and Analysis

High‐quality polysomes were purified using previously established protocols [78]. A 10%–50% (w/v) linear sucrose density gradient was prepared by freeze/thawing of a stepwise gradient from a series of 50, 40, 30, 20, and 10% sucrose buffers (20 mM Hepes pH 7.6, 100 mM KCl, 5 mM MgCl_2_, 1 mM DTT). The prepared gradients were thawed and equilibrated at 4°C overnight before use. Liver tissues collected from animals were lysed with hypotonic lysis buffer (5 mM Tris pH 7.5, 2.5 mM MgCl_2_, 1.5 mM KCl, 2 mM DTT, 100 µg/mL cycloheximide, 0.5% Triton X‐100, 0.5% sodium deoxycholate) by using Dounce homogenizer. RNase inhibitors were included to maintain RNA integrity. Lysed tissues were centrifuged for 2 min at 14 000 rpm. The supernatant was then loaded on the linear sucrose density gradient. The gradient tubes were the ultracentrifuged at 38 000 rpm for 2.5 h at 4°C (SW41 Ti rotor, Beckman). After ultracentrifugation, the gradient was fractionated into 15 equal parts using an automated density gradient fractionation system (Biocomp), while continuously recording the absorbance at 260 nm. RNA was extracted from each sucrose fraction, and the expression of target genes was analyzed by RT‐qPCR.

### 5.12 Isolation and Culture of Primary Hepatocytes

Mouse primary hepatocytes were isolated from mice using a two‐step collagenase perfusion procedure as previously described [79]. In brief, mouse was perfused via the portal vein with calcium‐free wash buffer (HBSS no Ca^2+^ no Mg^2+^, 25 mM HEPES, 0.5 mM EDTA, pH 7.4), and the vena cava was cut to wash out blood. Subsequently, liver was perfused with collagenase‐containing buffer (HBSS with Ca^2+^, Mg^2+^ and phenol red, 25 mM HEPES, pH 7.4, 0.7 mg/mL collagenase type I (Worthington, LS004196)) to loosen cell‐cell connections. The digested livers were minced to release cells, then filtered through a 70 µm cell strainer and centrifuged at 50 g for 2 min at 4°C. Cells were washed three times with cold plating medium, and then re‐suspended with 50% Percoll (GE Health) before centrifugation at 200 g for 10 min at 4°C. The pellet contains viable hepatocytes while dead cells and debris are left in the supernatant. The isolated mouse primary hepatocytes were cultured in collagen‐coated plates in DMEM containing 10% FBS and 1% antibiotics. After 4–6 h incubation, cells were treated with Tnfα (50 ng/mL) plus OA (500 µM) for 12 h to mimic the stimulation of free fatty acids and inflammatory cytokines during MASH progression.

### 5.13 Histology and Immunohistochemistry

Liver tissues were dissected and fixed in 4% paraformaldehyde and embedded in paraffin. According to standard protocols, paraffin embedding tissues were sectioned and subjected to H&E staining and Sirius red staining to assess morphologic changes and liver fibrosis. For detection of GPX1 (1:100, Abclonal, A23519), MSRB1 (1:100, Proteintech, 15333‐1‐AP), SBP2 (1:100, Abcam, Ab210791), αSMA (GB111364, Servicebio) and CD11B (GB11058, Servicebio), immunostaining staining was performed via standard protocols. Antigen retrieval was performed for 20 min in Tris/EDTA buffer (pH 9.0) or citrate buffer (pH 6.0) at 100°C. The HRP‐conjugated secondary antibody was used for signal detection (CST, 8125S). Immunohistochemistry‐stained tissue was visualized with Olympus IX73 microscopy.

### 5.14 Biochemical Assays

Whole blood samples were collected and allowed to clot at room temperature for 30 min. Serum was separated after centrifuging at 6000 rpm for 10 min. Serum ALT and AST were assessed using commercial kits (Shanghai Shensuo, 3040280 and 3050280) according to the manufacturer's instructions. All procedures were performed on ice. For lipid analysis in liver tissues, liver tissues were homogenized in ice‐cold PBS, and then mixed with chloroform: methanol (2:1) mixture for lipid extraction. The suspension was centrifuged at 2500 rpm for 10 min at 4°C, and the lipid fractions in the bottom organic phase were transferred to an empty tube, allowing to evaporate in a fume hood until dry. The residue was subsequently resuspended in 500 µL of 1% Triton X‐100 in ethanol for subsequent triglyceride or cholesterol measurement using triglyceride kit (Shanghai Shensuo, 1030280) or cholesterol kit (Shanghai Shensuo, 1040280). Lipid content was normalized to protein concentration.

### 5.15 Inductively Coupled Plasma‐Mass Spectrometry (ICP‐MS)

Selenium levels in serum and in liver tissue were determined according to the previously reported methods [80]. Briefly, serum was prepared by centrifugation at 2000 g for 15 min. Serum samples were then digested in 0.1% Trion X‐100+0.5% nitric acid. Lyophilized tissue samples were digested using a microwave‐assisted system prior to ICP‐MS analysis. Elemental analysis was performed using Agilent 7700X ICPMS. Serum selenium levels were expressed as a concentration and tissue selenium levels were normalized to the dry mass of tissue samples (g).

### 5.16 Detecting the Selenocysteine Incorporation Efficiency of Selenoprotein In Vivo by Bioluminescence Imaging

Based on the previously established selenocysteine (Sec) incorporation reporter assay [28], we developed an AAV‐delivered reporter to monitor the Sec incorporation efficiency, reflecting the selenoprotein translation efficiency in vivo. The GPx1 Sec‐insertion sequence (SECIS) element was amplified by PCR from mouse liver cDNA sample and cloned into the 3’ UTR of the luciferase reporter containing an in‐frame UGA codon at position 258. The resultant construct was designated Luc/UGA^258^/SECIS. For AAV vector construction, the GFP cassette in the AAV‐TBG‐GFP construct was replaced with Luc/UGA^258^/SECIS, for expression of luciferase with in‐frame UGA and SECIS, which mimics Sec incorporation. AAV‐TBG‐Luc and AAV‐TBG‐Luc/UGA^258^ were used as the positive or negative control. To evaluate the efficiency of Sec incorporation in this luciferase reporter system in vivo, 8‐weeks‐old male C57BL/6J mice were administered with (1) AAV‐TBG‐Luc, (2) AAV‐TBG‐Luc/UGA^258^, and (3) AAV‐TBG‐Luc/UGA^258^/SECIS via tail vein injection (2 × 10^12^ vector genomes per mouse). Bioluminescence imaging was performed in about two weeks after virus administration. To detect translation efficiency of selenoprotein in mice from NCD and MASH groups, mice were administered with AAV‐TBG‐Luc/UGA^258^/SECIS via tail vein injection (2 × 10^12^ vector genomes per mouse). Further experiments were subsequently begun in about two weeks after virus administration.

### 5.17 Luciferase Reporter Assay

The luciferase reporter assays were performed as described previously [81]. HEK293T cells were transfected with pGL4.11‐PPRE‐luc and pRL‐TK plasmid, in combination with indicated expression plasmids or empty vectors using polyethylenimine (PEI). After 12 h, the cells were treated with 10 µM wy‐14643 for 24 h. Luciferase activities were measured using the Dual Luciferase Reporter Assay System (Promega).

### 5.18 Immunofluorescence Staining

Cells were seeded on glass coverslips, fixed with 4% paraformaldehyde in PBS for 15 min, permeabilized with PBS containing 0.25% Triton X‐100 for 10 min, blocked with 1% BSA in PBS for 30 min (alternative blocking solutions are 10% serum) and incubated with rabbit polyclonal SELENOH antibody and mouse monoclonal PPARα antibody overnight at 4°C. After incubating with secondary antibodies and DAPI, images were captured using a Zeiss LSM880 confocal microscope. Fluorescence intensity was quantified using Image J software.

### 5.19 Liver FAO Activity Assay

Fatty acid oxidation (FAO) enzyme activities were measured using the FAO Assay Kit (Biomedical Research Service Center, E‐141) following the manufacturer's instructions. The assay is based on the oxidation of palmitoyl‐CoA, which is coupled to the NADH‐dependent reduction of INT to INT‐formazan, which has an absorption maximum at 492 nm. Liver tissue was homogenized in ice‐cold 1×Cell Lysis Solution, and the lysate was clarified by centrifugation. For each sample, 20 µg of protein was added in duplicate to a plain 96‐well plate. Then, 50 µL of control solution was added to one set of wells, and 50 µL of reaction solution was added to the other set of wells. The plate was later incubated in a non‐CO_2_ incubator at 37°C for 30 min, during which a cherry‐red color gradually developed in wells. The optical density (O.D.) reading of the control wells was subtracted from that of the reaction wells for each sample, with the resulting O.D. value proportional to the fatty acid oxidation activity of each sample.

### 5.20 FAOBlue Staining

FAOBlue is a fluorescent probe used to detect FAO activity in living cells [82]. It contains a nonanoic acid (C9) unit that undergoes metabolic degradation through sequential FAO enzyme reactions. In the final step, the intermediate would be hydrolyzed to release a fluorophore that emits bright fluorescence at 405 nm. Primary hepatocytes were isolated from control mice as well as *Selenoh* knockout or overexpression mice. Following Tnfα and OA treatment, primary hepatocytes were further treated with 10 µM FAOBlue for 30 min. Fluorescence imaging were performed using a Zeiss LSM880 confocal microscope and quantified using Image J software.

### 5.21 BODIPY Staining

Primary hepatocytes were stained with BODIPY 493/503 (GLPBIO, GC42959) to visualize lipid droplets. Cells were washed and stained with 2 µM BODIPY 493/503 in PBS for 15 min at 37°C. After washing, cells were fixed with 4% PFA at room temperature for 30 min. Nuclears were then marked with DAPI and cells were imaged using an Olympus IX73 microscopy. Fluorescence intensity was quantified using Image J software.

### 5.22 Molecular Docking

The tertiary structures of SELENOH and SELENOH^SXXS^ were predicted by Alphafold (https://alphafoldserver.com/). The protein structures of the PPARα ligand‐binding domain have been resolved, which was downloaded from the RCSB Protein Data Bank (https://www.rcsb.org/). Molecular docking was performed using the GRAMM Docking Web Server (https://gramm.compbio.ku.edu/request). PyMOL software was applied for analysis of the interaction interface residue between SELENOH and PPARα.

### 5.23 RNA Isolation and Real‐Time Quantitative PCR

Total RNA was isolated from liver tissues or cells by using TRIzol reagent (Thermo Fisher Scientific, 15596018) and then reverse transcribed with HiScript II Q RT SuperMix (Vazyme, R223‐01). The cDNAs were amplified by quantitative real‐time PCR using Taq Pro Universal SYBR qPCR Master Mix (Vazyme, Q712‐03) according to the manufacturer's protocol. The sequences of primers used are listed in Table S5.

### 5.24 RNA Sequencing Profiling

The mRNA sequencing was performed by Majorbio Company (Shanghai, China) and Genewiz from Azenta Life Sciences (Suzhou, China) according to the manufacturer's instructions. For data analysis, the raw RNA‐seq reads were aligned using the Hisat2, with mouse genome (GRCm38) as the reference genome. FeatureCounts was used to quantify gene abundances. We then used DESeq2 package in R software to identify differentially expressed genes (DEGs) with a cutoff of logFC > 1 and FDR adjusted *p* values ≤ 0.05. Gene Ontology (GO) analyses and Gene Set Enrichment Analysis (GSEA) were performed by using R package ClusterProfiler 4.0.

### 5.25 CUT&Tag‐Seq Analysis

CUT&Tag assays were performed by using Universal CUT&Tag Assay Kit (TD904, Vazyme) following the manufacturer's instructions. Briefly, liver tissue nuclei were isolated as previously described. The isolated nuclei were bound to concanavalin A‐coated magnetic beads, followed by incubation with primary and secondary antibodies. Hyperactive pA/G‐Transposon Pro complex was added to target the protein of interest under antibody guidance and fragment the chromatin DNA near the target protein. DNA from each sample was then purified and amplified for sequencing with the Illumina NovaSeq 6000. Raw sequence reads were mapped to the reference genome (GRCm38) by using Bowtie2. After removing adapter sequences and poor‐quality reads, the remaining reads were transformed by Samtools to generate BAM files. MACS2 was then used to call peaks with the IgG peaks as a control. Transcription factor binding motifs was further identified by HOMER software using default parameters. The bedgraph format files were generated using deepTools software for the visualization by IGV software. The primary antibodies used in CUT&Tag were SELENOH (NBP2‐14637, Novus Biologicals), PPARα (Ab227074, Abcam) and P300 (57625S, Cell signaling).

### 5.26 ATAC‐Seq Analysis

Nuclei were isolated from frozen liver tissue for ATAC‐seq following the previously described protocol [83]. Briefly, liver tissue was homogenized in homogenization buffer using the Dounce tissue homogenizer. After centrifugation to remove larger cellular debris from homogenized liver fluid, the supernatant containing nuclei was further purified by the gradient density centrifugation. About 100 000 purified nuclei were used to generate the ATAC‐seq library using the TruePrep DNA Library Prep Kit V2 for Illumina (Vazyme, TD501), which was then purified using the VAHTS DNA Clean Beads (Vazyme, N411), and sequenced with the Illumina NovaSeq 6000. Following the removal of adaptors, reads were aligned against reference genome GRCm38 by Bowtie2. We then filtered out reads that were unpaired, PCR duplicates, or mapped to multiple genomic sites. Reads with insertion length less than 100 bp were defined as fragments from nucleosome‐free regions, which were then used to call ATAC peaks by MACS2. Visualization of the ATAC‐seq data was accomplished by using Deeptools to generate bigwig format files.

### 5.27 scRNA‐Seq Analysis

Single cell RNA‐sequencing (scRNA‐seq) was performed at Singleron Biotechnologies (Nanjing, China). The raw sequencing data were aligned using the Cellranger software with the include‐intron alignment mode. After alignment, the Cellranger algorithm was applied to filter the expression matrix for downstream analysis by Seurat package. Initially, all cells were broadly categorized into three major groups: hepatocytes, immune cells, and stromal cells. Each of these groups was then individually re‐clustered and manually annotated for sub‐populations. Within the immune cell population, cells were first divided into lymphoid and myeloid lineages, followed by unsupervised clustering to identify distinct immune cell types. When comparing the two groups, DEGs for each cell type were calculated, with the threshold set as log_2_FC ≥ 0.5 and q‐value < 0.1. Clustering of DEGs in hepatocytes was performed by using pheatmap package, with the number of clusters set to six and the clustering arguments set to default values. GO enrichment analysis for specific gene clusters was conducted by using clusterProfiler package. To assess the overall impact of the selenium supplement on gene expression in hepatocytes of MASH model, the Findmarkers function from the Seurat package was used to identify DEGs of hepatocytes in the MASH group compared to the NCD group. Genes with significantly reduced expression in MASH group were defined as “Downregulated_Hepatocyte_Genes MASH_vs_NCD,” which was then used as a reference gene set for GSEA of DEGs in MASH + Se group compared to MASH group.

### 5.28 Generation of Human Liver Organoids

To develop HLOs, human induced pluripotent stem cells (hiPSC) were differentiated into foregut, which were then cultured in 3D with retinoid acid (RA), followed by further maturation in liver maturation medium as previously described [45]. Briefly, the 1016 hiPSC clone was cultured to 80%–90% confluence prior to differentiation. On day 1, cultures were transitioned to RPMI 1640 with 100 ng/mL activin A and 50 ng/mL BMP4, followed by medium replacement on day 2 with RPMI 1640/100 ng/mL activin A/0.2% FBS, and then transitioned on day 3 to RPMI 1640/100 ng/mL activin A/2% FBS. During days 4–6, cells were maintained in Advanced DMEM/F12 supplemented with 1×N2, 1×B27, 1×GlutaMax, 10 mM HEPES, 1% Penicillin‐Streptomycin, 500 ng/mL FGF4, and 3 µM CHIR99021. On Day 7, foregut cells were dissociated using Accutase into single cells, and embedded in Matrigel at 1 × 10^5^ cells/100 µL. Matrigel‐embedded foregut cells were cultured in Advanced DMEM/F12 containing 5 ng/mL FGF2, 10 ng/mL VEGF, 20 ng/mL EGF, 3 µM CHIR99021, 0.5 µM A83‐01, and 50 µg/mL ascorbic acid, changing the medium every 2 days. On day 11, the culture medium was adjusted to Advanced DMEM/F12 containing 2 µM retinoic acid for 4 days, changing the medium every 2 days. Final maturation step (days 15–21) utilized hepatocyte culture medium containing 100 nM dexamethasone, 20 ng/mL oncostatin M, 10 ng/mL HGF, and 10% Matrigel, with medium changed every 3 days.

### 5.29 Steatohepatitis Modeling in HLOs

After 6 days of maturation, HLOs were subjected to steatohepatitis modeling. Briefly, HLOs were treated with pre‐cooled PBS to remove the surrounding Matrigel, followed by resuspension in HCM complete medium with 500 µM OA and TGFβ. After 3 days of culture, HLOs were harvested for downstream analyses.

### 5.30 Statistics

All results were presented as mean ± SEM. Replicates are indicated in figure legends. n represents the number of experimental replicates. Statistical comparisons between two groups were made by the unpaired Student's *t*‐test, and for comparison of more than two groups, one‐way ANOVA with post hoc Bonferroni's multiple comparison test was adopted, as indicated in the figure legends. The correlation between selenium and metabolic parameters was performed using Spearman's correlation analysis. *p* values were calculated in GraphPad Prism 8.0 (GraphPad Software, LaJolla, CA, USA), which can be found in the individual figure panels and figure legends.

## Author Contributions

Q.D. and Y.Z. conceived and designed the project; Y.Z., YC.W., B.L., X.L., C.L., Y.C., C.T., D.W., X.G., and YD.W. performed the experiments and analyzed data; Y.Z., YD.W., and Q.D. wrote the manuscript; Q.D., YD.W,. and C.J. supervised the project.

## Funding

This work was supported by Program of EnShi Tujia&Miao Autonomous Prefecture Bureau of Scientific & Technological Affairs, the National Key R&D Program of China (2023YFA1801100), Noncommunicable Chronic Diseases‐National Science and Technology Major Project (2023ZD0507700), the National Natural Science Foundation of China (92357306, 32200629, 82330028, 82425013, 32300999, 32300994), the Strategic Priority Research Program of the Chinese Academy of Sciences (XDB1150201), the China Postdoctoral Science Foundation (2023M733632, 2023M733633), Changzhou Medical Center of Nanjing Medical University Program (CMCM202403), the Key R&D Program of Shandong Province (2025CXPT176), the Research Project of Jinan Microecological Biomedicine Shandong Laboratory (JNL2025008B, JNL2025009B, JNL2025010B, JNL2025011B), the Taishan Scholar Young Expert Program of Shandong Province (tsqn202507393, 202408310), and the Youth Innovation Promotion Association of CAS.

## Ethics Statement

All animal experiments in this study were performed according to protocols approved by the Institutional Animal Care and Use Committee of the Shanghai Institute for Nutrition and Health (ethical committee approval no.SINH‐2022‐DQR‐1). All experiments involving patients were approved by the Science and Technology Ethics Committee of Linyi People's Hospital in accordance with the principles of the Declaration of Helsinki (202501‐H‐005).

## Consent

Written informed consent was obtained from all human participants prior to the research.

## Conflicts of Interest

The authors declare no conflicts of interest.

## Supporting information




**Supporting File**: advs74266‐sup‐0001‐SuppMat.docx.

## Data Availability

All data are available in the main text or the supplementary materials. All biological materials used are readily available from the authors or from standard commercial sources. The source data required to reanalyze the data included in this report are available from the corresponding author upon request. Sequencing data (RNA‐seq, CUT&Tag, ATAC‐seq, scRNA‐seq) have been deposited in NODE (http://www.biosino.org/node): OEP00006620 and OEP00006618.
