# Supplementary material for: Selenoprotein H Functions as a PPARα Coactivator to Link Selenium Homeostasis to Hepatic Lipid Metabolism and Protect against Steatohepatitis
Source: Adv Sci (Weinh). 2026 Feb 8;13(22):e19563. doi: 10.1002/advs.202519563 (PMC13088273; doi:10.1002/advs.202519563)
Supplement: Supplementary file 1 — Supporting File: advs74266‐sup‐0001‐SuppMat.docx. [file ADVS-13-e19563-s001.docx]

**Selenoprotein H functions as a PPARα coactivator to link selenium homeostasis to hepatic lipid metabolism and protect against steatohepatitis**

Yuwei Zhang^1^, Yuchen Wang^1^, Binbin Li^2^, Xin Li^3^, Chenyu Liu^1^, Yanhao Chen^1^, Cheng Tian^1^, Dongmei Wang^4^, Xiaosong Gu^3,5^, Chunping Jiang^3,5,6*^, Yuda Wei^7*^, Qiurong Ding^1,8,9*^

**Table of contents**

Supplementary Fig.1-11

Supplementary Table.1-5

**Supplemental figures and tables**


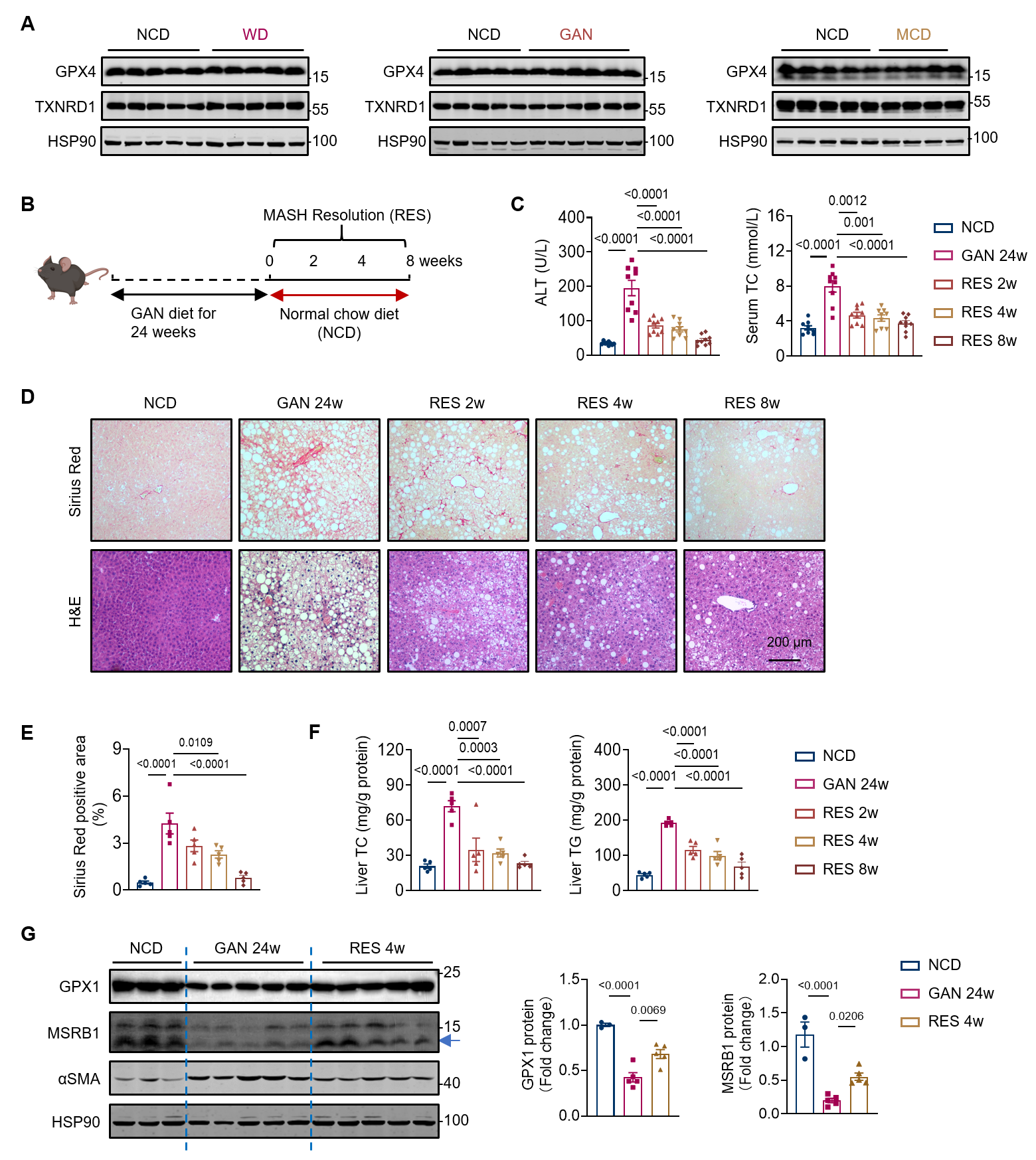


**Figure S1. Expression of selenoproteins after MASH resolution. Related to Figure 1.**

1. Protein levels of GPX4 and TXNRD1 in the livers from different mouse models of MASH.
2. Schematic of the experimental design: for inducing MASH resolution, GAN diet-fed mice were switched to a normal chow diet (NCD) for 2, 4, 8 weeks.
3. Blood ALT and total cholesterol (TC) levels of animals as indicated. n = 9 per group.
4. Representative H&E and Sirius Red staining of liver sections from animals as indicated. Scale bars, 200 μm.
5. Percent (%) Sirius Red positive area measured on ImageJ. n = 5 per group.
6. Hepatic TC and triglyceride (TG) levels of animals as indicated. n = 9 per group.
7. Protein levels of GPX1, MSRB1 and αSMA in the livers from animals as indicated.

Values are mean ± SEM. The one-way ANOVA with post hoc Bonferroni multiple-comparison test (C, E-G) was used for statistical analysis.


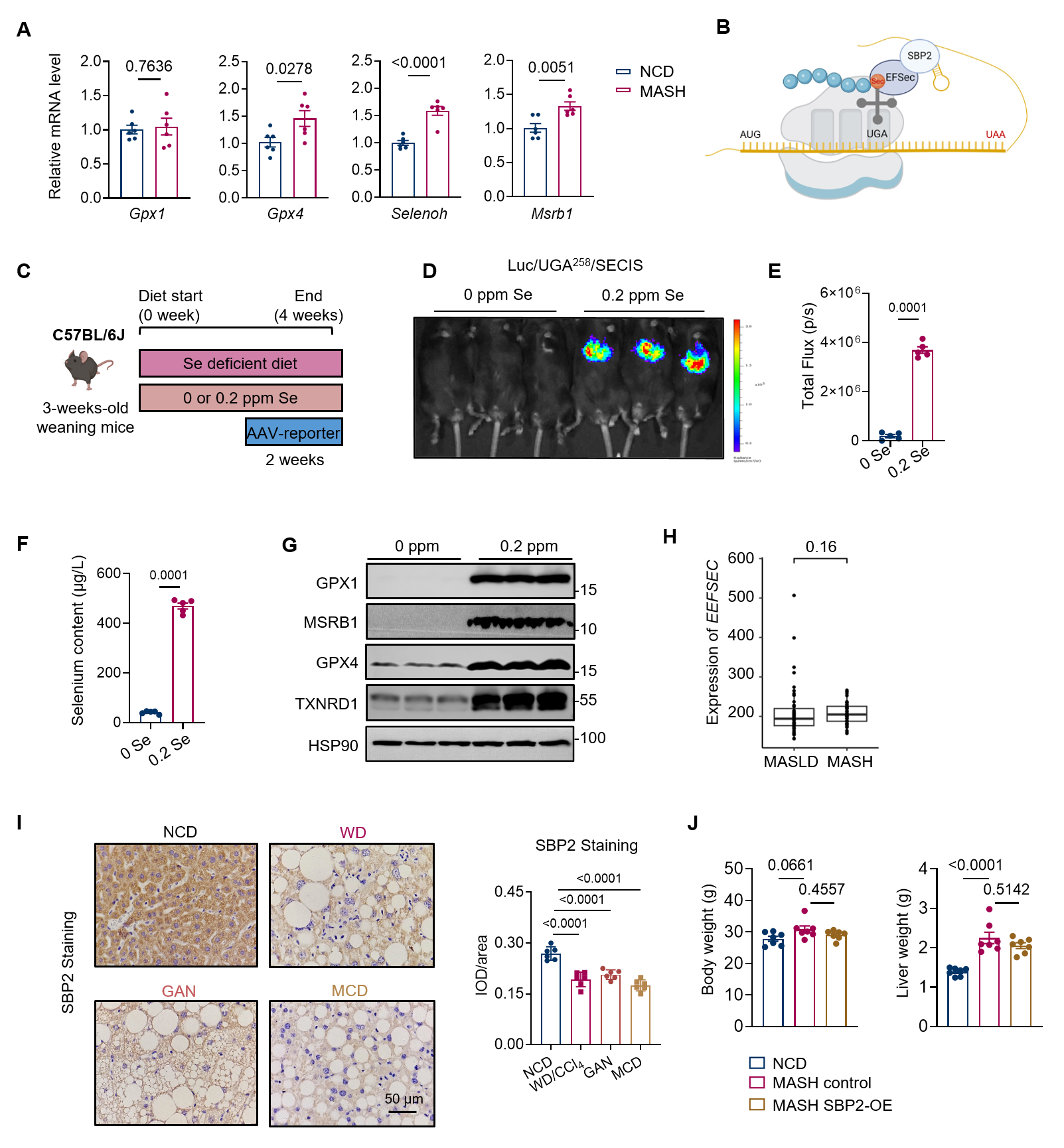


**Figure S2. MASH livers exhibit impaired selenoprotein synthesis. Related to Figure 2.**

1. Relative mRNA levels of *Gpx1*, *Gpx4*, *Selenoh*, and *Msrb1* between NCD and WD-induced MASH livers. n = 6 per group.
2. Schematic illustration of selenocysteine insertion mechanism.
3. Schematic of the experimental design: 0 ppm and 0.2 ppm selenium treatment on weaning mice.
4. Representative images after injection with AAV expressing luciferase/UGA^258^/SECIS in mice fed with 0 ppm and 0.2 ppm selenium.
5. Normalized quantification of bioluminescent imaging in (D). n = 5 per group.
6. Selenium levels in serum from mice fed with 0 ppm and 0.2 ppm selenium. n = 5 per group.
7. Protein levels of selenoproteins GPX1, MSRB1, GPX4 and TXNRD1 in the livers. n = 3 per group.
8. *EEFSEC* mRNA expression in FPKM from GSE167523 [MASLD (n = 51) and MASH (n = 47)].
9. Representative SBP2 staining of liver sections from animals as indicated. n = 6 per group.
10. Body and liver weights in NCD, MASH control and MASH SBP2 overexpresion (OE) mice, n = 7 per group.

Values are mean ± SEM. The unpaired Student’s t test (A, E, F), wilcoxon test (H) and one-way ANOVA with post hoc Bonferroni multiple-comparison test (I and J) were used for statistical analysis.


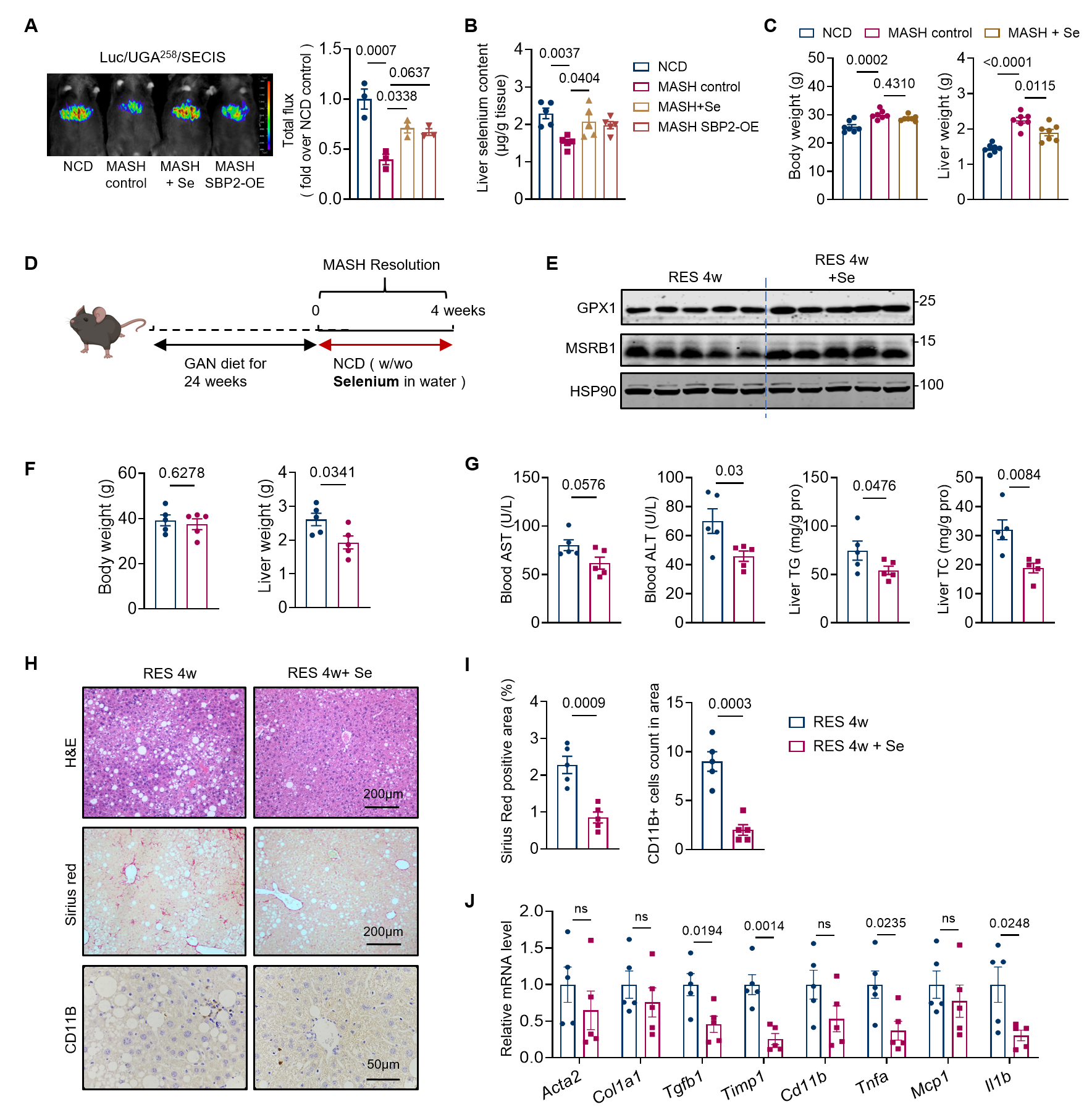


**Figure S3. Dietary selenium supplementation promotes MASH resolution and fibrosis regression. Related to Figure 3.**

1. Representative image and normalized quantification of bioluminescent imaging after injection with AAV expressing luciferase/UGA^258^/SECIS in mice as indicated. n = 3 per group.
2. Selenium levels in liver tissue from mice as indicated. n = 5 per group.
3. Body and liver weights in NCD, MASH control and MASH selenium (Se) supplementation mice, n = 7 per group.
4. Schematic of the experimental design: MASH resolution (RES) mice were treated with selenium in water (RES 4w + Se group) or vehicle (RES 4w group) for 4 weeks after the diet switch.
5. Protein levels of GPX1 and MSRB1 in the livers from animals as indicated.
6. Body and liver weights in animals as indicated, n = 5 per group.
7. Blood AST and ALT levels, and hepatic TG and TC levels of animals as indicated. n = 5 per group.
8. Representative H&E, Sirius Red and CD11b staining of liver sections from animals as indicated.
9. Percent (%) Sirius Red positive area and CD11b+ cells count based on the immunohistochemistry analysis (E). n = 5 per group.
10. Relative mRNA levels of genes related to hepatic inflammation and fibrosis. n = 5 per group.

Values are mean ± SEM. The one-way ANOVA with post hoc Bonferroni multiple-comparison test (A-C) and unpaired Student’s t test (F, G, I and G) were used for statistical analysis.


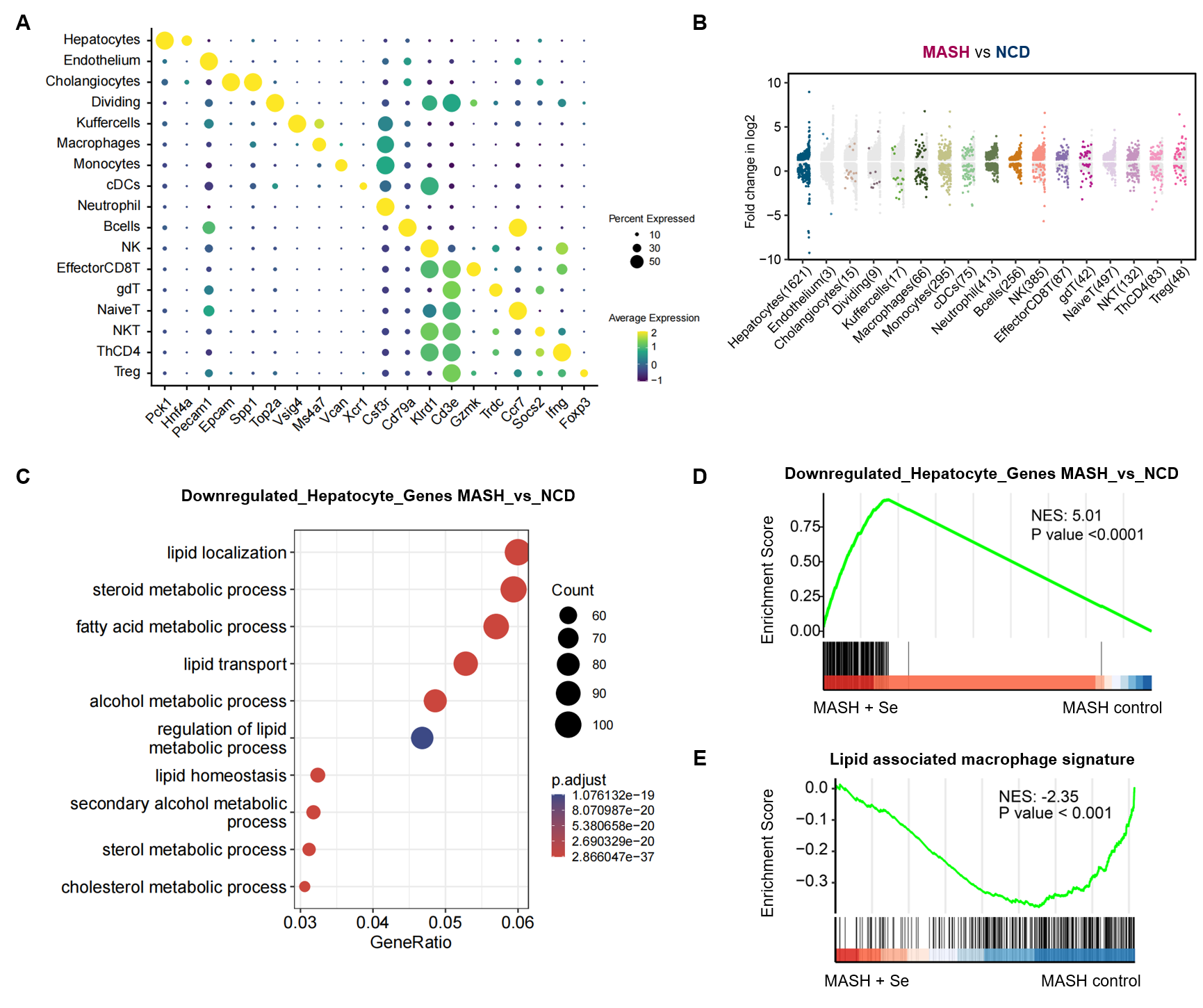


**Figure S4. Single-cell RNA-seq analysis of the effects of selenium supplementation on various cell types in MASH liver.** **Related to Figure 3.**

1. Dot plot showing the expression of marker genes of manually annotated cell type.
2. Dot plot showing the fold changes of genes in each cell type. Genes with positive values have higher expression levels in MASH group, while those with negative values have higher expression levels in NCD group. Genes with adjusted *P* value < 0.01 are shown with indicated colors, while genes with insignificant changes between groups are shown in grey dots. The number in parenthesis showing the number of significantly changed genes.
3. Dot plot showing the GO analysis results of downregulated genes of hepatocytes in MASH group compared to NCD group.
4. GSEA analysis of differentially expressed genes of hepatocytes in MASH + Se group compared to MASH control group. Downregulated genes analysed in (C) are used as the reference gene list.
5. GSEA analysis of differentially expressed genes of macrophages in MASH + Se group compared to MASH control group. Lipid associated macrophage (LAM) signature is referred to GSE128518.


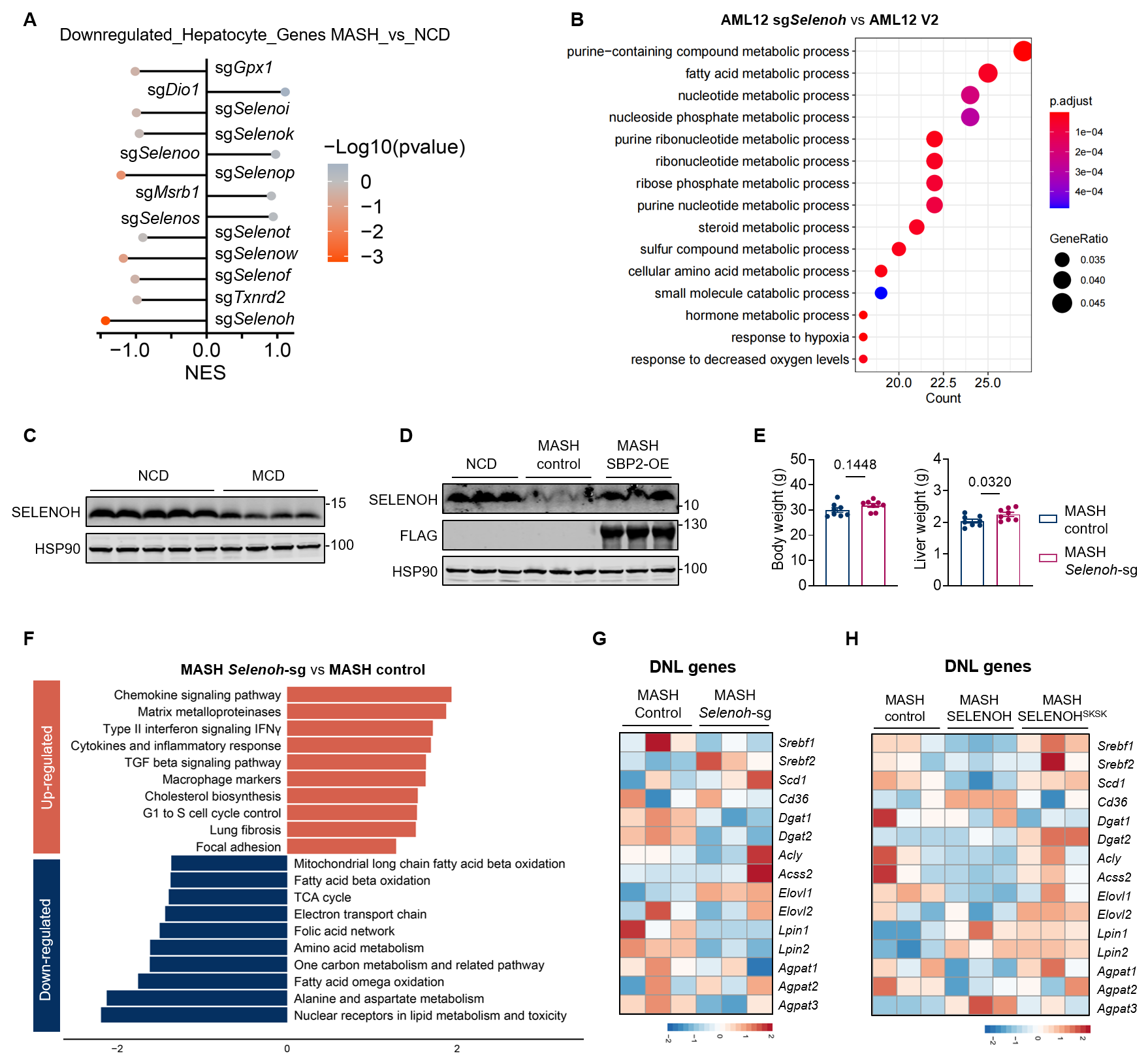


**Figure S5. The transcriptomic changes after *Selenoh* knockout most closely resemble the downregulated gene set in MASH vs. NCD. Related to Figure 4.**

1. Lollipop chart showing the normalized enrichment scores from GSEA analysis of differentially expressed genes in each indicated group against downregulated genes of hepatocytes in MASH group vs NCD group by scRNA-seq.
2. Dot plot showing the GO analysis results of downregulated genes in AML12 *Selenoh*-sg group compared to the control group.
3. Protein levels of SELENOH in the livers from NCD (n=5) and MCD-induced MASH (n=5) mice.
4. Protein levels of SELENOH in the livers from NCD, MASH control and MASH SBP2 overexpression (OE) mice. n = 3 per group.
5. Body and liver weights in MASH control and MASH *Selenoh*-sg mice, n = 5 per group.
6. GSEA analysis of upregulated and downregulated genes in livers of MASH *Selenoh*-sg group compared to MASH control group.
7. Heatmap representing the expression of genes associated with *de novo* lipogenesis (DNL) pathways in MASH and MASH *Selenoh*-sg livers.
8. Heatmap representing the expression of genes associated with DNL pathways in MASH control, MASH SELENOH-OE and MASH SELENOH^SKSK^-OE mice livers.

Values are mean ± SEM. The unpaired Student’s t test (E) was used for statistical analysis.

**
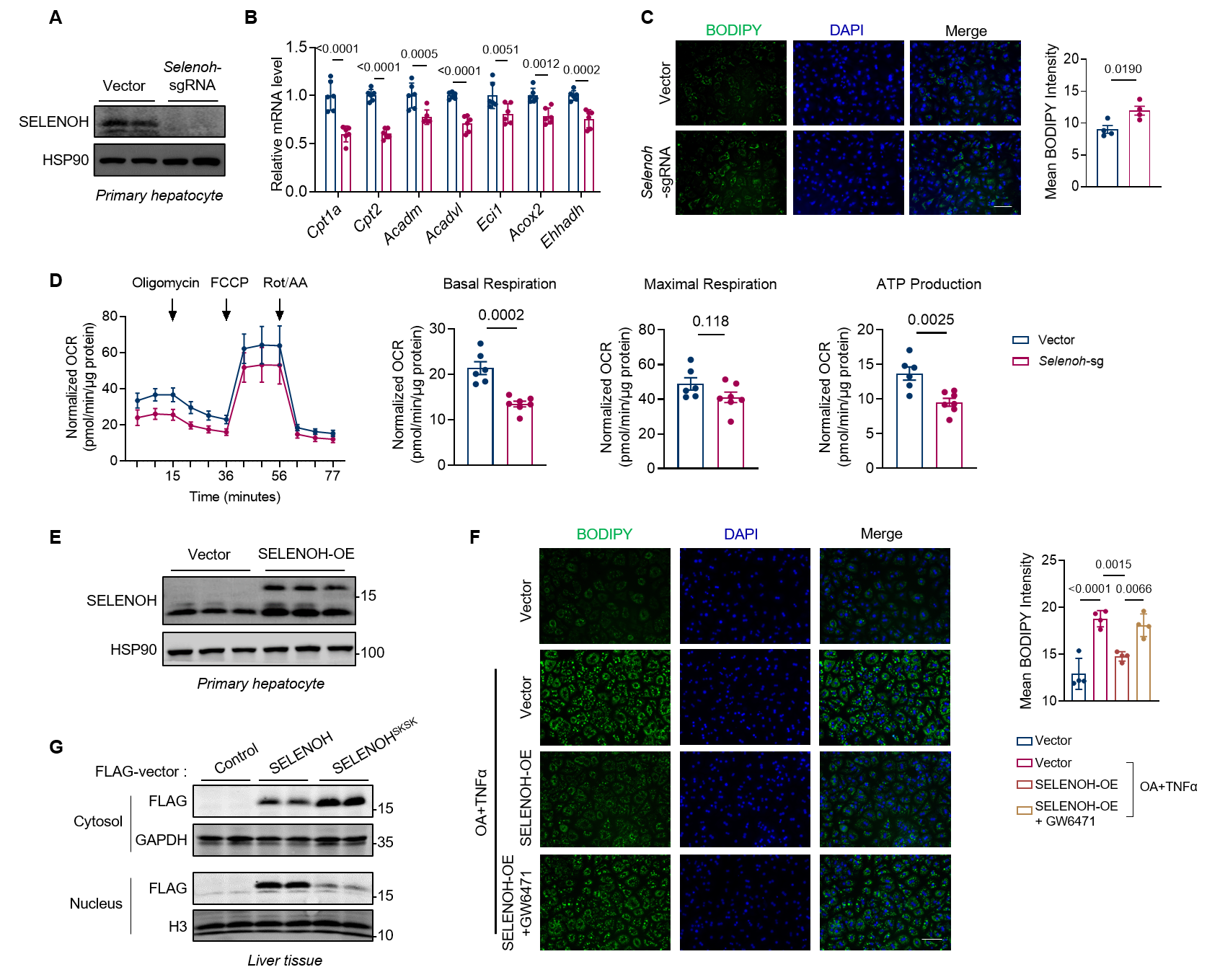
**

**Figure S6. Examination of fatty acid oxidation in primary hepatocytes. Related to Figure 4.**

1. Protein levels of SELENOH in primary hepatocytes from mice injected with AAV-TBG-control or AAV-TBG-*Selenoh*-sgRNA.
2. Relative mRNA levels of indicated FAO genes in primary hepatocytes from mice injected with AAV-TBG-control or AAV-TBG-*Selenoh*-sgRNA. n = 6 per group.
3. Representative images of lipid accumulation in primary hepatocytes from mice injected with AAV-TBG-control or AAV-TBG-*Selenoh*-sgRNA. Cells were stained with BODIPY for fat accumulation (green) and DAPI for the nucleus (blue). Scale bars, 100 μm.
4. Oxygen Consumption Rate (OCR) was measured in primary hepatocytes from mice injected with AAV-TBG-control or AAV-TBG-*Selenoh*-sgRNA using the Seahorse XFe96 analyzer. Quantification of basal respiration, ATP production and maximal respiration. Control n = 6; *Selenoh*-sgRNA, n = 7.
5. Protein levels of SELENOH in primary hepatocytes from mice injected with AAV-TBG-control or AAV-TBG-SELENOH. n = 3 per group.
6. Representative images of lipid accumulation in primary hepatocytes from mice injected with AAV-TBG-control or AAV-TBG-SELENOH. Primary hepatocytes were treated with 10 μm GW6471 (PPARα-selective antagonist) for 16 hours. Cells were stained with BODIPY for fat accumulation (green) and DAPI for the nucleus (blue). Scale bars, 100 μm. n = 3 per group.
7. Western blot analysis showing the protein levels of FLAG-tagged SELENOH or SELENOH^SKSK^ in the nucleus and cytoplasm of liver tissue from indicated mice.

Values are mean ± SEM. The unpaired Student’s t test (B-D) and one-way ANOVA with post hoc Bonferroni multiple-comparison test (F) were used for statistical analysis.

**
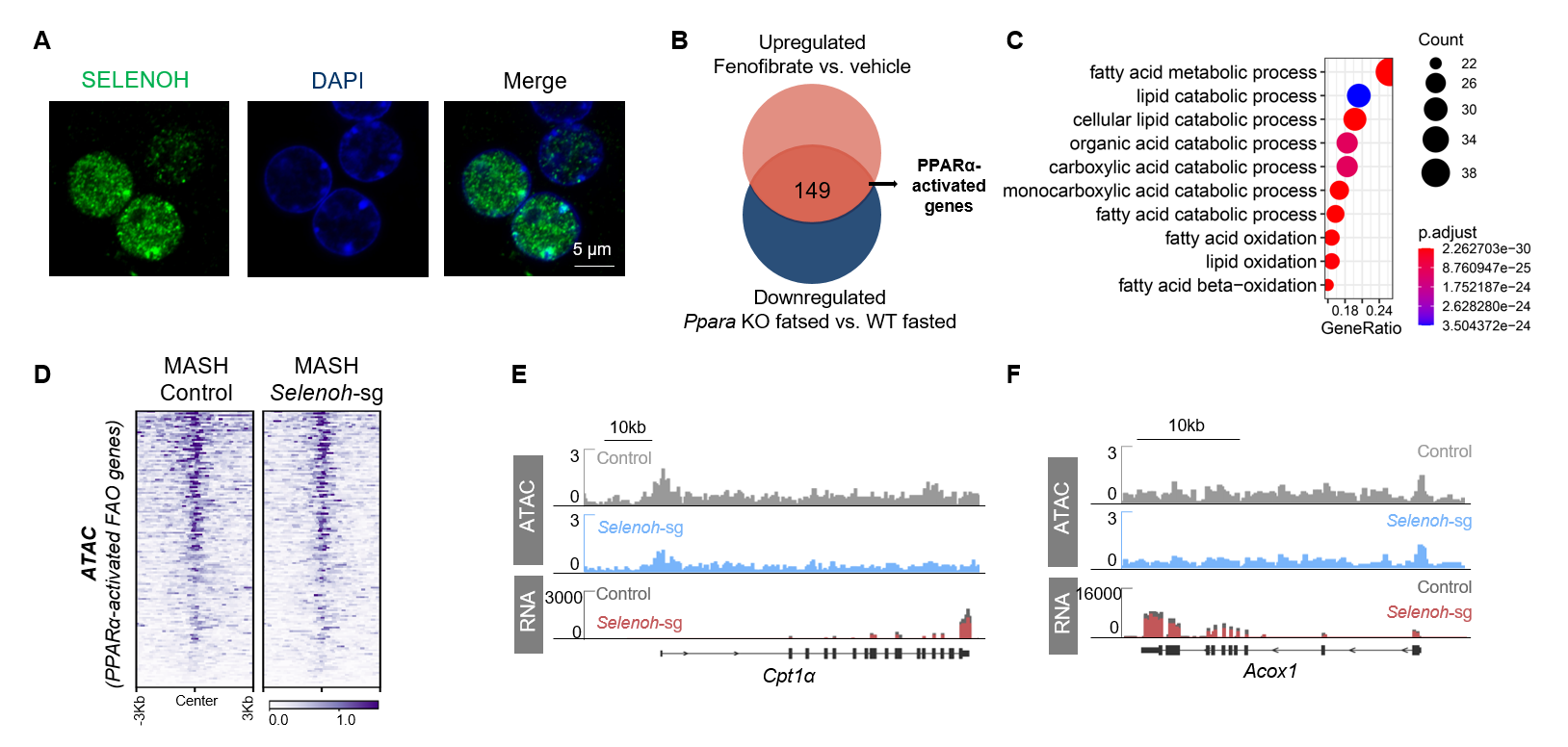
**

**Figure S7. SELENOH regulates fatty acid oxidation via PPARα. Related to Figure 5.**

1. Representative immunofluorescence of SELENOH in primary hepatocytes. Scale bars, 5 μm.
2. Flowchart representing the comparative analysis of transcriptomic data using different datasets (GSE73298 and GSE73299).
3. Dot plot showing the GO analysis results of PPARα-activated genes in (B).
4. Heatmap showing ATAC-seq signals at PPARα-activated genes in MASH control and MASH *Selenoh*-sg livers.

(E and F) ATAC-seq and RNA-seq tracks of *Cpt1a* and *Acox1* loci.


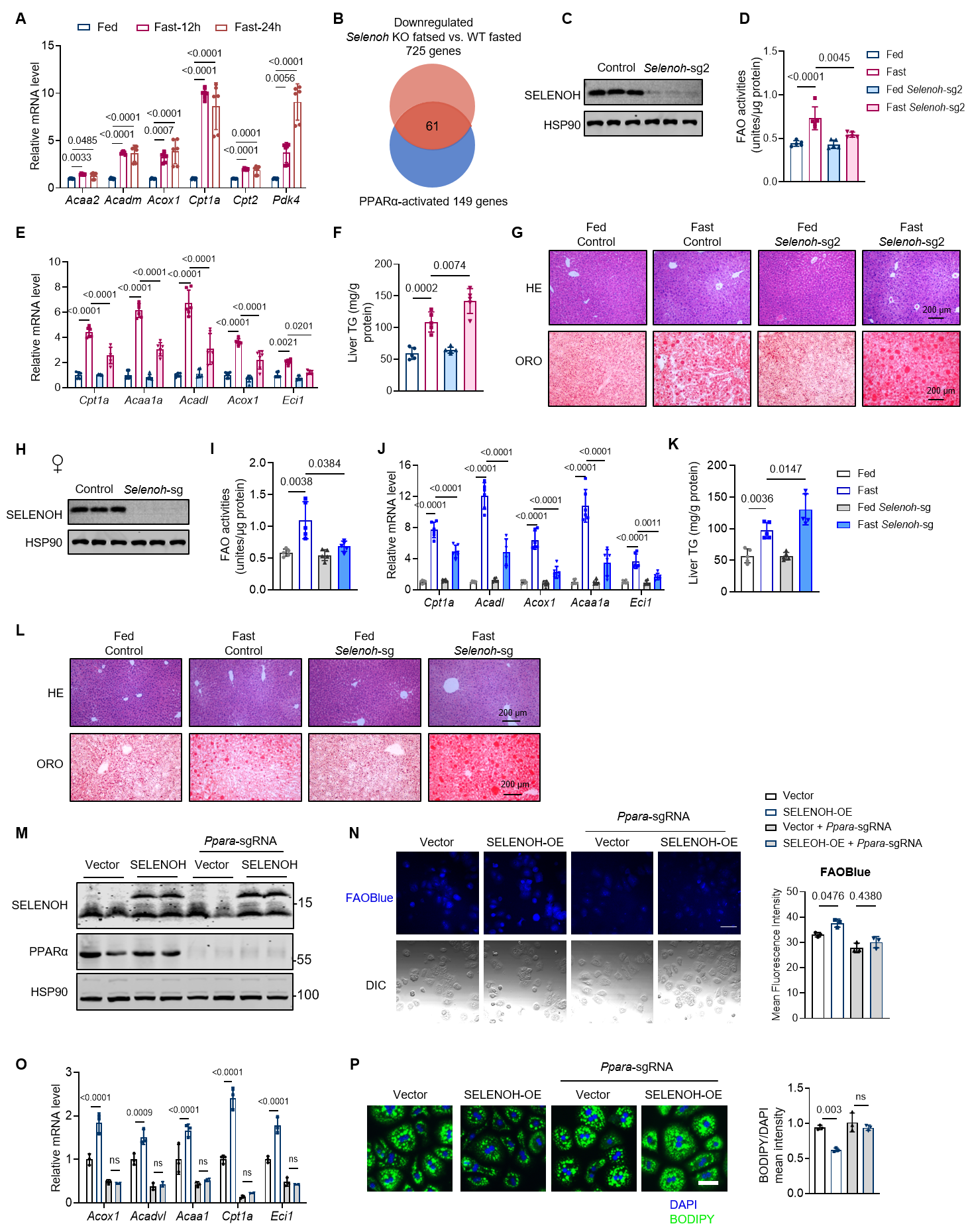


**Figure S8. SELENOH regulates fasting-induced fatty acid oxidation. Related to Figure 5.**

(A) Relative mRNA levels of indicated FAO genes in liver tissues from fast and fed animals. n = 3 per group.

(B) Comparative analysis of PPARα-activated FAO genes and *Selenoh* liver-specific KO transcriptomes.

(C) Protein level of SELENOH in control and SELENOH-depleted (via *Selenoh*-sgRNA-2) livers.

(D) FAO activities of liver tissues.

(E) Relative mRNA levels of indicated FAO genes in control and SELENOH-depleted livers.

(F) Hepatic TG levels of animals as indicated.

(G) Representative H&E and Oil Red O staining of liver sections from animals as indicated.

(H) Protein level of SELENOH in control and SELENOH-depleted livers of female mice.

(I) FAO activities of liver tissues from female mice in different groups.

(J) Relative mRNA levels of indicated FAO genes in control and SELENOH-depleted livers from female mice.

(K) Hepatic TG levels of female animals as indicated.

(L) Representative H&E and Oil Red O staining of liver sections from female animals as indicated.

(M) Protein levels of SELENOH and PPARα in primary hepatocytes from indicated mice.

(N) Representative images and quantification of FAOBlue in primary hepatocytes. Cells were stained with FAOBlue (blue) to detect FAO activity. Scale bars, 100 μm.

(O)Relative mRNA levels of indicated FAO genes in primary hepatocytes from indicated mice. n = 3 per group.

(P)Representative images of lipid accumulation in primary hepatocytes from indicated mice. Cells were stained with BODIPY for fat accumulation (green) and DAPI for the nucleus (blue). Quantification by ImageJ normalized to DAPI intensity. n = 3 independent culture experiments. Scale bars, 50 μm.

Values are mean ± SEM. The one-way ANOVA with post hoc Bonferroni multiple-comparison test (G, J-L) was used for statistical analysis.

**
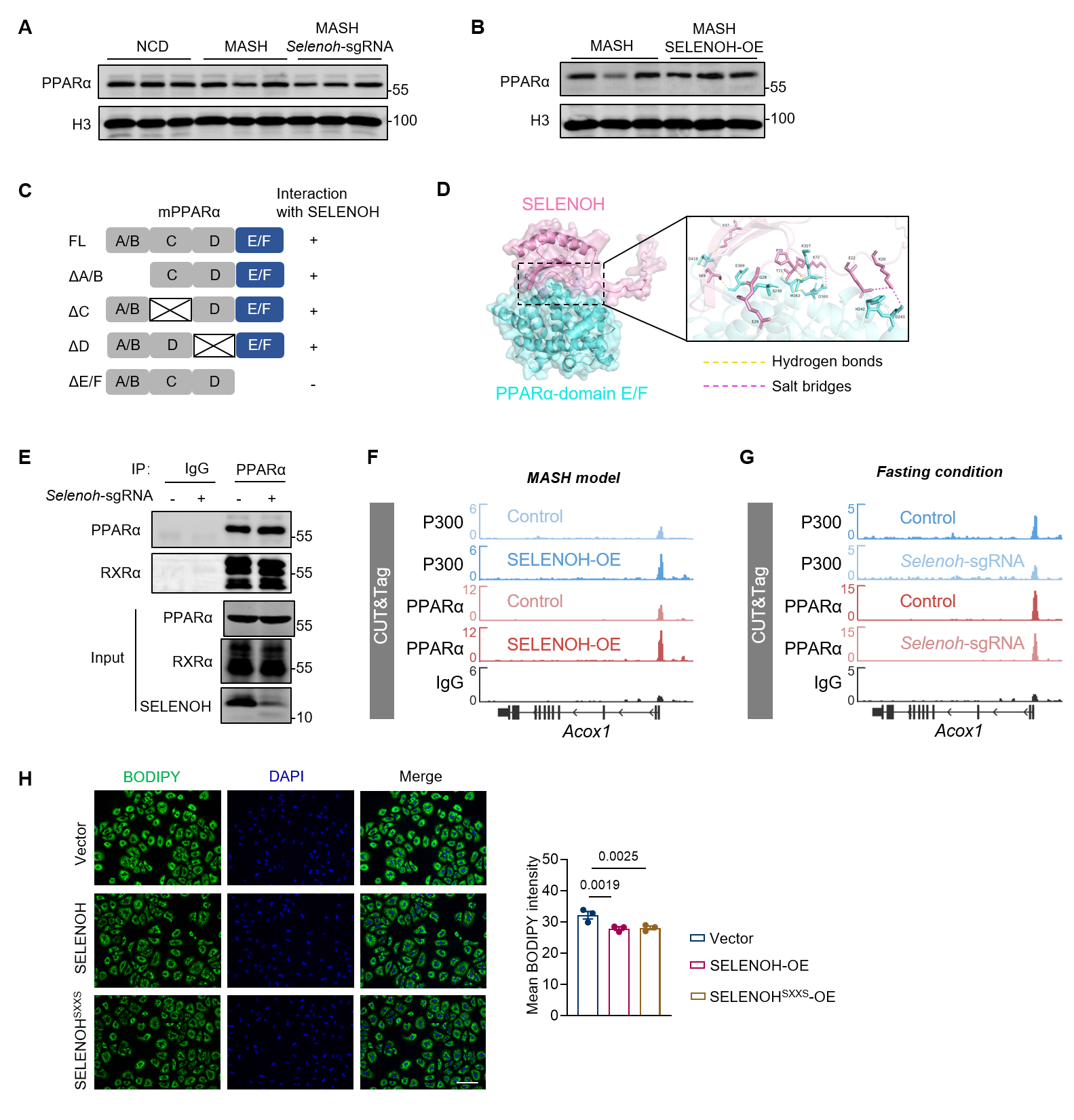
**

**Figure S9. SELENOH interacts with PPARα to enhance its genomic binding in a manner independent of SELENOH's antioxidant activity. Related to Figure 6.**

(A and B) Protein levels of PPARα in the livers from indicated mice.

1. E/F domain is the major PPARα domain mediating SELENOH interaction.
2. Molecular docking of SELENOH and E/F domain of PPARα.
3. Endogenous co-immunoprecipitation of PPARα and RXRα in liver nuclear proteins from mice injected with AAV-TBG-control or AAV-TBG-*Selenoh*-sgRNA under the fasting condition.

(F and G) CUT&Tag tracks of P300 and PPARα peaks at *Acox1* locus.

(H) Representative images of lipid accumulation in primary hepatocytes from indicated mice. Cells were stained with BODIPY for fat accumulation (green) and DAPI for the nucleus (blue). Scale bars, 100 μm.


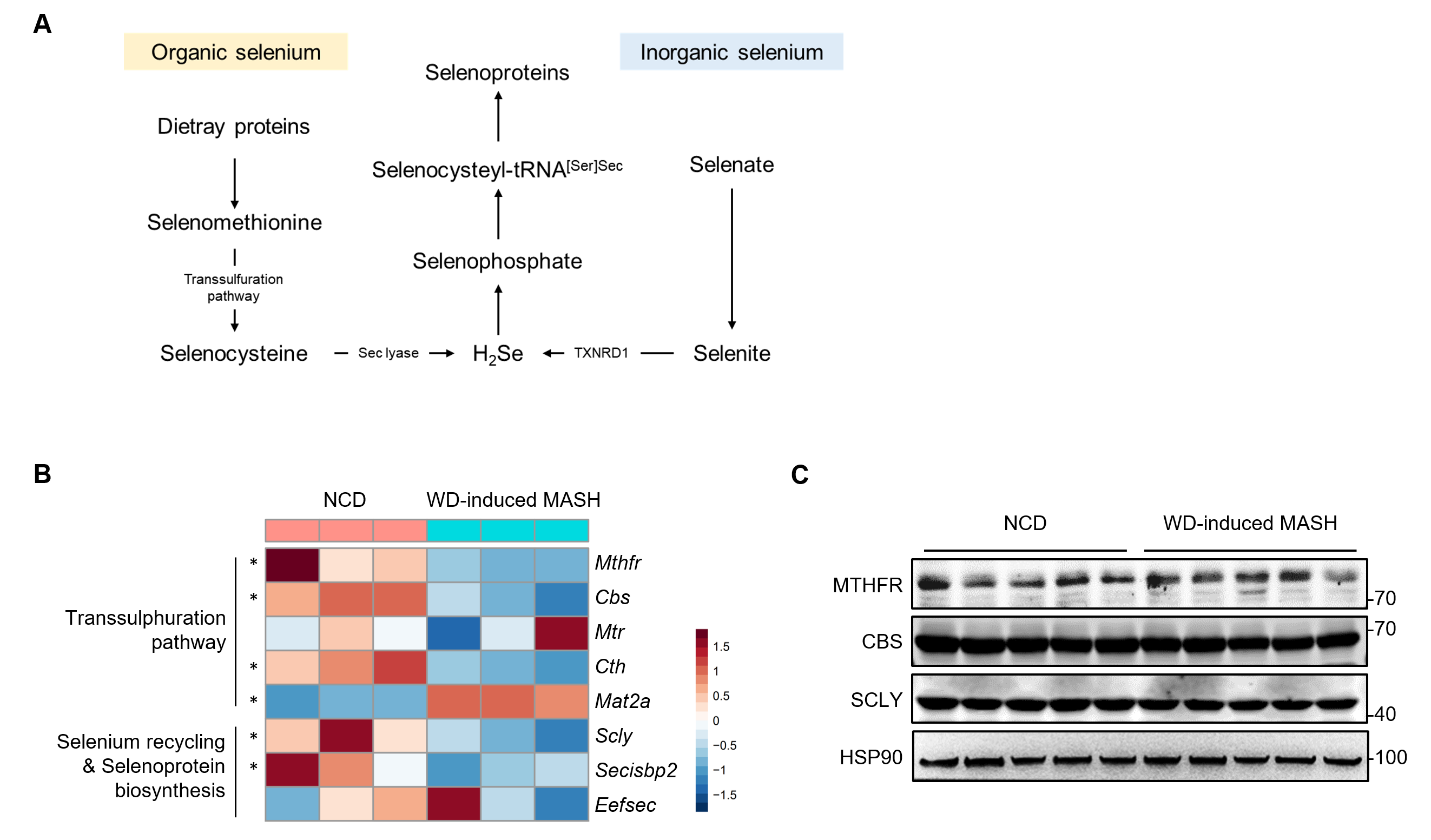


**Figure S10. Selenomethionine metabolism and selenium recycling remain unaffected in MASH.**

1. Schematic illustration of selenium metabolism.
2. Heatmap showing the expression of indicated selenium recycling genes in liver samples from NCD and WD-induced MASH models.
3. Protein levels of selenium recycling genes in the livers from NCD and WD-induced MASH models.

**
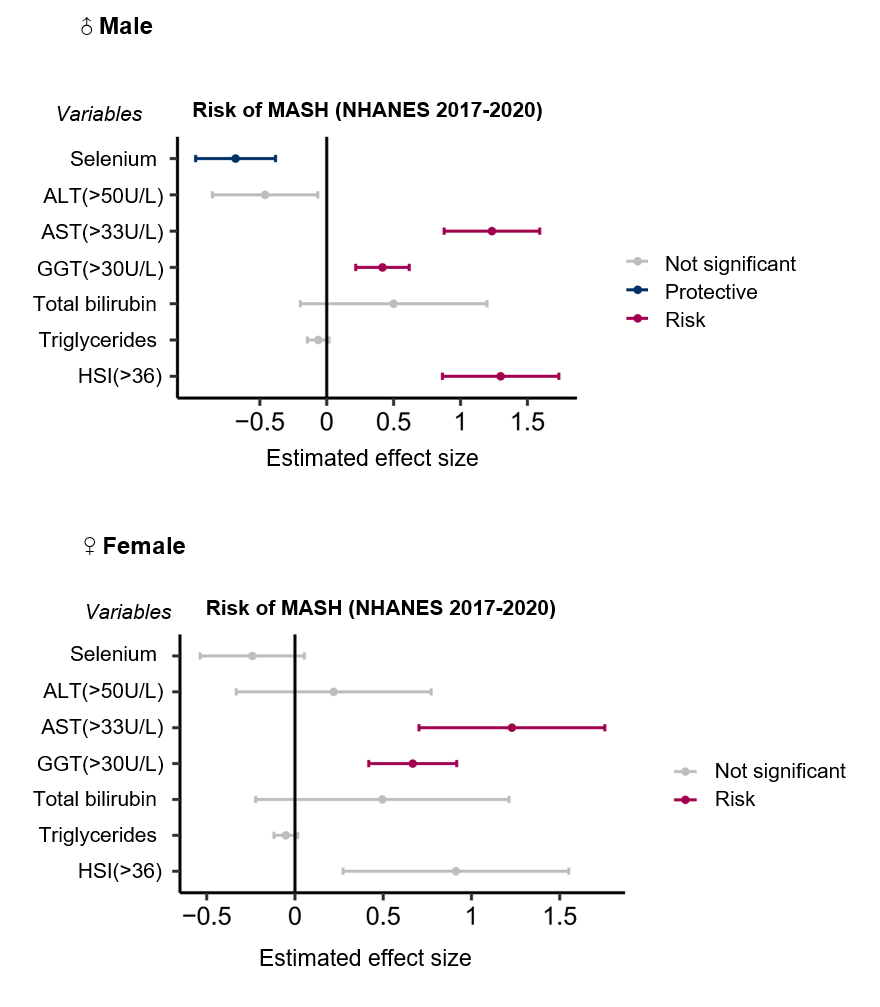
**

**Figure S11. Multivariable logistic regression of risk factors for MASH development.** Sex-Stratified analysis of MASH risk in clinical cohort 2 (data from NHANES 2017-2020) by multi-variate logistic regression analysis.

**Table S1.** Selenoproteins with relatively high expression levels in liver.

|  | Selenoprotein | Genename | Function |
| --- | --- | --- | --- |
| 1 | Glutathione peroxidase 1 | Gpx1 | Cytosolic glutathione peroxidase |
| 2 | Iodothyronine deodinase 1 | Dio1 | Thyroid hormone-activating  iodothyronine deodinase |
| 3 | Selenoprotein I | Selenoi | Unknown |
| 4 | Selenoprotein K | Selenok | Putative role in ER-associated degradation |
| 5 | Selenoprotein O | Selenoo | Unknown |
| 6 | Selenoprotein P | Selenop | Se transport |
| 7 | Methionine-R-sulfoxide reductase | Msrb1 | Reduction of oxidized methionine  residues |
| 8 | Selenoprotein S | Selenos | Putative role in ER-associated degradation |
| 9 | Selenoprotein T | Selenot | Unknown |
| 10 | Selenoprotein W | Selenow | Unknown |
| 11 | Selenoprotein F | Selenof | Putative role in quality control of protein folding in the ER |
| 12 | Thioredoxin reductase 2 | Txnrd2 | Reduction of mitochondrial thioredoxin and glutaredoxin |
| 13 | Selenoprotein H | Selenoh | Unknown |

**Table S2.** sgRNA sequences used in the experiments.

| **Target Gene** | **sgRNA Target Sequence** |
| --- | --- |
| *Gpx1* | GCTCGAACCCGCCACCAGGT |
| *Dio1* | GCGGCTTGTGATATTCCTGC |
| *Selenoi* | AGTTTTCGGGTCGTCATGGC |
| *Selenok* | TAATTCTGTAGGTCAGGTGT |
| *Selenoo* | AGCGCGAGACCGACGCACCG |
| *Selenop* | TCAGCAATGTGGAGAAGCCT |
| *Msrb1* | AGTACGCACACTCATCCCCG |
| *Selenos* | GCCTCAAAGCCCTCAGTCGA |
| *Selenot* | GTCGGCGGTGGTCCGCAGCG |
| *Selenow* | AGCTTGAGGCTATAAGCCCA |
| *Selenof* | GCTGGCGACTGCGTTTCAAG |
| *Txnrd2* | CGTGTCAGAGCCCGTGTCCG |
| *Selenoh-1* | CGGAAGAAAGCGTAAGGCGG |
| *Selenoh-2* | AGTACGAGCTGACGCGTGTA |
| *Ppara* | CATCGAGTGTCGAATATGTG |
| *Ep300* | GCGGCCTAAACTCTCATCTC |

**Table S3.** Clinical characteristics of human subjects in clinical cohort 1.

| Variables | Healthy (n = 15 ) | Hepatic steatosis  (n = 15) | *P* value |
| --- | --- | --- | --- |
| Age (year) | 35.14 ± 6.467 | 40 ± 8.92 | 0.1069 |
| Gender (female, n) | 7 | 4 | / |
| BMI (kg/m2) | 21.64 ± 1.421 | 32.12 ± 9.89 | 0.0005 |
| Total cholesterol (mmol/L) | 4.389 ± 0.3621 | 5.053 ± 1.028 | 0.0255 |
| Triglycerides (mmol/L) | 1.003 ± 0.3513 | 2.434 ± 1.385 | 0.0006 |
| ALT (U/L) | 15.68 ± 8.044 | 109.3 ± 54.6 | <0.0001 |
| AST (U/L) | 19.08 ± 3.3 | 58.01 ± 37.6 | 0.0004 |
| HSI | 28.49 ± 1.314 | 48.54 ± 10.22 | <0.0001 |
| Diabetes (n) | 0 | 2 | / |

**Table S4.** Primer sequences used to construct plasmids in the experiments.

| Selenoh-SXXS-F | tggtcattgagcattctacgagctcacgcgtgtacggccg |
| --- | --- |
| Selenoh-SXXS-R | cggccgtacacgcgtgagctcgtagaatgctcaatgacca |
| Selenoh-SKSK-F | catTCTAGAatggccccccacggaagcaagagtaaggcg |
| Selenoh-SKSK-R | catGGATCCacaatgtcttgctggagggctgcctcacaactg |
| Ppara-ΔA/B-F | catTCTAGAtgtcgaatatgtgggg |
| Ppara-ΔA/B-R | catGGATCCgtacatgtctctgtag |
| Ppara-ΔC-F | gtcatacatgacatggagaccttgtgtatg |
| Ppara-ΔC-R | catacacaaggtctccatgtcatgtatgac |
| Ppara-ΔD-F | ctgtctgtcgggatggagaccttgtgtatg |
| Ppara-ΔD-R | catacacaaggtctccatcccgacagacag |
| Ppara-ΔE/F-F | CATTCTAGAatggtggacacagaga |
| Ppara-ΔE/F-R | catGGATCCcatgtcatgtatgaca |

**Table S5.** RT-qPCR primers used in the experiments.

| *Gapdh*-qPCR-F | AGGTCGGTGTGAACGGATTTG |
| --- | --- |
| *Gapdh*-qPCR-R | TGTAGACCATGTAGTTGAGGTCA |
| *Gpx1*-qPCR-F | AGTCCACCGTGTATGCCTTCT |
| *Gpx1*-qPCR-R | GAGACGCGACATTCTCAATGA |
| *Msrb1*-qPCR-F | CTTCGGAGGCGAGGTTTTCC |
| *Msrb1*-qPCR-R | TCTCAGGGCACTTGGTCACA |
| *Cd11b*-qPCR-F | CTCACGTATCCGTGCCTTCTT |
| *Cd11b*-qPCR-R | GTCCACGCAGTCCGGTAAA |
| *Mcp1*-qPCR-F | TTAAAAACCTGGATCGGAACCAA |
| *Mcp1*-qPCR-R | GCATTAGCTTCAGATTTACGGGT |
| *Tnfa*-qPCR-F | TATGGCTCAGGGTCCAACTC |
| *Tnfa*-qPCR-R | CTCCCTTTGCAGAACTCAGG |
| *Il1b*-qPCR-F | GCCCATCCTCTGTGACTCAT |
| *Il1b*-qPCR-R | AGGCCACAGGTATTTTGTCG |
| *Timp1*-qPCR-F | ACTCGGACCTGGTCATAAGGG |
| *Timp1*-qPCR-R | CGCTGGTATAAGGTGGTCTCGT |
| *Col1a1*-qPCR-F | GAACTGGACTGTCCCAACCC |
| *Col1a1*-qPCR-R | TTGGGTCCCTCGACTCCTAC |
| *Acta2*-qPCR-F | CAGCCATCTTTCATTGGGATGGAG |
| *Acta2*-qPCR-R | AATGCCTGGGTACATGGTGG |
| *Tgfb1*-qPCR-F | GCCCGAAGCGGACTACTATG |
| *Tgfb1*-qPCR-R | ATAGATGGCGTTGTTGCGGT |
| *Selenoh*-qPCR-F | TGGACAAGCGCGAGAAACTG |
| *Selenoh*-qPCR-R | CAGCTCGTACAATGCTCAATGA |
| *Cpt1a*-qPCR-F | TGGCATCATCACTGGTGTGTT |
| *Cpt1a*-qPCR-R | GTCTAGGGTCCGATTGATCTTTG |
| *Cpt1b*-qPCR-F | GCACACCAGGCAGTAGCTTT |
| *Cpt1b*-qPCR-R | CAGGAGTTGATTCCAGACAGGTA |
| *Cpt2*-qPCR-F | CAGCACAGCATCGTACCCA |
| *Cpt2*-qPCR-R | TCCCAATGCCGTTCTCAAAAT |
| *Acadvl*-qPCR-F | CTACTGTGCTTCAGGGACAAC |
| *Acadvl*-qPCR-R | CAAAGGACTTCGATTCTGCCC |
| *Acadl*-qPCR-F | TCTTTTCCTCGGAGCATGACA |
| *Acadl*-qPCR-R | GACCTCTCTACTCACTTCTCCAG |
| *Acadm*-qPCR-F | CCAGAGAGGAGATTATCCCCG |
| *Acadm*-qPCR-R | TACACCCATACGCCAACTCTT |
| *Eci1*-qPCR-F | GCAGGGGTTGCAGTGATGAA |
| *Eci1*-qPCR-R | GATGACACCTCGGATGCTCTT |
| *Acaa1a*-qPCR-F | TCTCCAGGACGTGAGGCTAAA |
| *Acaa1a*-qPCR-R | CGCTCAGAAATTGGGCGATG |
| *Acaa2*-qPCR-F | CTGCTACGAGGTGTGTTCATC |
| *Acaa2*-qPCR-R | AGCTCTGCATGACATTGCCC |
| *Acox1*-qPCR-F | TAACTTCCTCACTCGAAGCCA |
| *Acox1*-qPCR-R | AGTTCCATGACCCATCTCTGTC |
| *Acox2*-qPCR-F | AACCCAGGGGATCGAGTGT |
| *Acox2*-qPCR-R | CGCAGCTCAGTGTTTGGGAT |
| *Ehhadh*-qPCR-F | ATGGCTGAGTATCTGAGGCTG |
| *Ehhadh*-qPCR-R | GGTCCAAACTAGCTTTCTGGAG |
